# Explaining building damage from wildfires in California

**DOI:** 10.1126/sciadv.aed4197

**Published:** 2026-07-24

**Authors:** Somnath Bar, Shu Li, Tirtha Banerjee

**Affiliations:** ^1^ Department of Civil and Environmental Engineering, University of California, Irvine, Irvine 92697, USA.; ^2^ Department of Earth System Science, University of California, Irvine, Irvine 92697, USA.

## Abstract

A physically interpretable, data-driven framework was developed to elucidate causal interactions, model, and predict wildfire-induced building damage across California. More than 100,000 damage inspection records (2013 to 2024) were used to model building damage from static environmental variables (topography, vegetation, and human footprint), dynamic weather inputs, and a proposed Composite Building Flammability Rating (CBFR). Three model configurations were tested: (i) a comprehensive model integrating all variables, (ii) an enviro-weather hybrid excluding CBFR, and (iii) an environmental exposure model excluding both weather and CBFR. A strict 200-meter spatial dead-zone constraint was applied to eliminate local autocorrelation, and the comprehensive model achieved 88% (±0.4%) accuracy, which dropped to 82.9% (±0.6%) without CBFR and to 74.5% (±0.5%) without both weather and CBFR. Spatial grid-based cross-validation demonstrated a diverse accuracy of 68.0 (±17%), 66.0 (±16%), and 62.0 (±13%), respectively. Building flammability, dew point temperature, and near-surface wind speed were identified as the most important predictors of damage. Vapor pressure deficit had the strongest causal effect on damage probability, though spatial variability was observed in the causal effects of climate and geographic variables. A 100-meter-resolution Wildfire Building Damage Risk Index was also developed to highlight high-risk damage zones. Findings emphasize that wildfire impacts in the wildland-urban interface result from a confluence of structural vulnerability, atmospheric dryness, and fuel exposure, offering scalable tools for risk forecasting, defensible space planning, and climate-resilient infrastructure development.

## Teaser

Wildfire building damage is fundamentally driven by a compounding confluence of structural vulnerability, dryness, and wind.

## INTRODUCTION

Recent decades have witnessed a sharp rise in both the size and destructiveness of wildfires in California, particularly since 2000, leading to unprecedented property damage and loss of life, particularly in the wildland-urban interface (WUI) regions of the state (*1*, *2*). In the United States (US), WUI areas have expanded by about 33% between 1990 and 2010, adding more than 12 million additional structures (*3*). Although WUI expansion slowed slightly in the 2010s compared with the 2000s, ∼30% of all new homes built between 2011 and 2020 were located in the WUI in the US, down from 39% during 2001 to 2010 (*4*). Between 2020 and 2022, more than 80% of new homes in California were built in high fire-risk areas (*5*). Rapid residential growth combined with larger, more severe burned areas has substantially increased the vulnerability of communities, as many of the newly built homes are now situated in high-risk WUI zones (*6*, *7*). Most of the building damage from wildfires is observed within the WUI, where human-made structures are interspersed with wildland vegetation (*7*–*9*). This growth, combined with more frequent and extensive wildfires, has doubled the number of houses burned within wildfire perimeters since the 1990s. Approximately 47% of this destruction was due to housing growth in the wild lands, while 53% was due to increased fire activities (*10*). California has witnessed more than 55,000 houses burned or destroyed between 2010 and 2024 (*11*). As risk increases, it is essential to accurately assess the influence of factors ranging from individual building characteristics to broader landscape-scale exposures to inform adaptive mitigation strategies, land-use planning, and resilience-building efforts.

As of 2022, ∼28,575 km^2^ of land in California was classified as WUI, comprising 56% intermix (developments scattered within with wildland vegetation) and 44% interface (developments adjacent to contiguous wildland areas) WUI area. Within the intermix, 52.3% consists of grassland and shrubland, while only 30.1% is forest area. Notably, 79.5% of destroyed buildings in the western US are lost to grassland and shrubland fires, and the remainder are lost to forest fires (*10*). The presence of fine fuels, such as grass and shrubs, near structures in areas with a frequent fire history and low-to-intermediate structural density increases this risk by promoting rapid fire spread and ignition (*12*). The climate-driven changes in plant functional types (PFT) are projected to shift 40% (under Representative Concentration Pathway 4.5; RCP4.5) to 58% (under RCP8.5) of the current forested area in the western US to shrublands and grasslands by 2070 to 2100 (*13*). Therefore, the fuel distribution or PFT proportions around the building, along with the type, height, and coverage of vegetation, are critical parameters that affect flame length, fire intensity, and rate of spread (*14*, *15*). In addition to the fuel type, the local topography and ecosystem gradient collectively set the boundary conditions for fire propagation. Several studies, using data-driven and physics-based models, have identified that topographic slope, elevation, and aspect are important factors influencing fire behavior and building damage (*7*, *16*–*18*). In California, a combination of fuel aridity and prolonged periods of extreme weather, especially hot, dry, and windy conditions, has quadrupled the number of conditions conducive to large wildfires since the late 20th century (*19*, *20*).

Extreme fire weather often plays a substantial role in large-scale damage in California, particularly during Santa Ana winds, which can rapidly escalate fire intensity and spread (*21*). The Palisades and Eaton Fires in 2025, which destroyed more than 16,000 structures, illustrate the devastating potential of these extreme weather events (*22*–*24*). The firebrands, or ember particles, are products of extreme fire weather and often serve as the immediate cause of building ignitions in or near the WUI. However, the structural type and materials of a building ultimately determine its flammability once embers land (*25*, *26*). Studies have found that roof materials, exterior, eave types, materials of vent screens, and deck materials notably influence building flammability (*7*, *8*, *19*, *20*, *27*, *28*). Moreover, embers can ignite nearby flammable vegetation, enabling the fire to spread and intensify around the building, ultimately increasing the likelihood of structural damage. A probabilistic, data-driven framework grounded in causal inference could be a pragmatic approach to exploring the complex interactions among terrain, ecosystems, and fire weather that drive wildfire damage across diverse landscapes.

Remote sensing data, particularly granular-scale Earth Observation (EO) data, provide opportunities to explore wildfire damage mechanisms by incorporating gridded environmental, weather, human footprint, and building flammability-level information. The development and application of machine learning (ML) and deep learning techniques to analyze Earth-surface processes (*29*) have advanced substantially over recent decades, and the integration of rich cubes of EO data has further expanded their potential across multiple scientific domains, particularly in wildfire science, spanning from early detection (*30*, *31*) and risk assessment (*7*) to postfire impact analysis (*32*, *33*), like infrastructural damage. Intuitively, traditional ML techniques are effective for risk analysis and damage prediction; however, they provide limited insight into how the driving factors interact causally to produce the observed damage outcomes. On the other hand, causal learning enables the exploration of “what-if” scenarios, estimating how changes in key variables would influence outcomes (*34*). The integrated use of traditional ML and causal learning provides an effective framework to unravel the interactions, risks, and predictive factors of wildfire-related infrastructure damage at both building and landscape scales. Previous studies have explored these relationships at the landscape level. For example, several studies (*12*, *28*, *35*) used generalized linear models to assess the influence of vegetation, topography, building characteristics, and spatial arrangement on wildfire damage. They found that landscape features such as connectivity, topography, and the spatial arrangement of buildings were strong predictors of building loss. However, they did not account for the dynamic weather conditions. Another study by Dossi *et al.* (*8*) proposed a Wildfire Resistance Index for building vulnerability to wildfire based solely on construction features. Similarly, Papathoma-Köhle *et al.* (*7*) developed a statistically validated physical vulnerability index that identified critical indicators (e.g., roof material, structural type, and topographic slope) while disproving the relevance of others, such as neighboring buildings in dense settlements. Chulahwat *et al.* (*36*) demonstrated that graph-theoretic metrics of building vegetation interactions can quantify building vulnerability, although their approach assumes static fire perimeters and uniform wind conditions. A recent study by Zamanialaei *et al.* (*37*) used ML to predict structure losses in WUI from five large fires (2017 to 2020) but relied on reconstructed fire exposure, lacked direct flame-scale measurements, and omitted fire-weather variables. Furthermore, these studies are constrained by overreliance on static, generalized risk factors; lack of regional adaptability; small sample sizes; fire event–specific analyses; and the omission of key environmental, weather, and fire-behavior variables.

Here, we address these gaps by integrating traditional ML approaches and causal learning using more than 100,000 postfire building damage observations (2013 to 2024) with static environmental factors, dynamic fire weather, human footprint, and building features, focusing on building- and community-level damage from wildfires. We compiled an extensive dataset of building inspections from 2013 to 2024, capturing structural losses across diverse fire events in California. These data are combined with finer-resolution (30-m) information on fuels, topography, and daily weather conditions (∼4 km) at each building location and its surroundings. The study operates on the central assumption that building damage results from the interplay between fire behavior and building vulnerability, where environmental conditions, human footprint, and weather dynamics modulate fire behavior, including ember transport mechanisms. The overarching objectives of the study are (i) to unfold the key static and dynamic environmental drivers and their quantified scale of contribution to wildfire-induced building damage across California; (ii) to assess the relative influence and causal effects of building flammability, fire weather components, fine fuel (grass/shrub) exposure, and fraction in shaping wildfire damage potential using a data-driven model; and (iii) to develop a building damage risk matrix, integrate it with WUI, and produce a dynamic WUI-risk map to inform targeted wildfire mitigation strategies.

## RESULTS

### Wildfire building damage along the environmental gradient

**Fig. 1.**
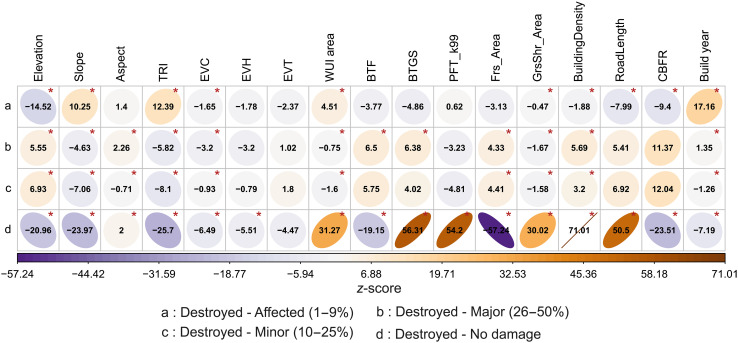
Significant group differences across building damage classes. *z*-scores from Dunn’s post hoc tests comparing destroyed (damaged) and other building damage classes across the explanatory variables. Colored ellipses represent the direction and magnitude of group-wise differences (*z*-score), with red asterisks indicating statistically significant differences (P<0.05).

The analysis of building damage data revealed that 86% of damaged buildings are located within the WUI, encompassing both interface and intermix areas. According to the building damage scale, 54% (53,879) of postfire inspected buildings are destroyed, and 41% (40,895) are undamaged from 22 fires (2013 to 2024), (fig. S1 and table S1). Furthermore, the distribution of static environmental factors, including elevation, slope, aspect, terrain ruggedness, ecosystem variables, and human footprints, varied significantly across different wildfire building damage classes. Probability density analysis (fig. S2) along with Kruskal-Wallis and Dunn’s tests (Fig. 1 and Table 1) demonstrated that destroyed buildings (>50% damage) were predominantly associated with mid-elevations (522.00 m, ±370.32 m), gentle to moderate slopes (5°; $\pm 4.75^{\circ }$), and lower terrain roughness (TRI = 7.21;±6.9). While the topographic aspect showed weaker overall differentiation (Kruskal-Wallis $\chi^{2}=24.7$, P<0.05). Destroyed structures exhibited slightly lower northness (0.038±0.771) and more negative eastness (−0.097 ±0.629) than no-damaged buildings (0.080±0.774 and −0.053 ±0.626, respectively), with circular mean aspects of 292° (west-northwest) versus 327° (north-northwest), representing a ∼35° westward shift. However, the high circular SDs (∼2.1 rad) indicate that buildings sustain damage across all orientations under extreme fire conditions. The damage classes vary significantly (<0.05) across vegetation composition. The destroyed buildings were predominantly situated in open-canopy, low-to-moderate density vegetation areas characterized by shrubs and grasses (heights ≤1 to 3 m), as well as in forested areas with trees ranging from 5 to 25 m in height. These vegetation types mainly included California Annual Grassland, California Mesic Chaparral, and Mediterranean California Mixed Oak Woodland, followed by California Montane Woodland and Chaparral, and California Mixed Evergreen Forest (Table 1 and Fig. 1). Wildfires disproportionately destroyed high-WUI areas (median = 100% WUI), while low-WUI zones remained mostly intact (P<0.001). The average ${\text{PFT}}_{k9}$ (proportion of woody and fine fuel) in damaged (>50) buildings has 24% grass/shrub dominance.

**Table 1.** Nonparametric rank differences predictor variables across building damage classes. Results of Kruskal-Wallis rank sum tests assessing the variability of each variable across building damage classes. All tests yielded statistically significant results (P<0.001), indicating potential influence of each variable on damage outcomes. BTF, building–to–forest area ratio; BTGS, building–to–grassland area ratio in 100-m grid.

Variable	Kruskal-Wallis ($\chi^{2}$)	*P* value
Elevation	901.87	<0.001
Slope	648.74	<0.001
Aspect	24.67	<0.001
EVC	102.67	<0.001
EVH	94.13	<0.001
EVT	40.52	<0.001
TRI	781.48	<0.001
WUI area (km^2^) in 100-m grid	2021.91	<0.001
BuildingDensity	5437.60	<0.001
BTF	557.14	<0.001
BTGS	3315.04	<0.001
Frs_Area	3587.26	<0.001
GrsShr_Area	999.71	<0.001
RoadLength	2622.99	<0.001
${\text{PFT}}_{k9}$	3173.90	<0.001
Build year	386.84	<0.001
CBFR	1241.27	<0.001

Dunn’s post hoc tests revealed that the most significant divergence occurred between the destroyed and no-damage buildings across all variables. The strongest negative divergence was observed in the forest area (Frs_Area; *z*-score = −57.24, P<0.01), followed by TRI, slope, and elevation, respectively, indicating that the destroyed group had a lower median compared with no-damage buildings. In contrast, strong positive differences were found in building density (*z*-score = 71.01, P<0.01), followed by the building-to-grass/shrub ratio (BTGS), ${\text{PFT}}_{k9}$, road length, and WUI area within a 100-m grid, suggesting that the destroyed group had a higher median than the no-damage buildings. On the other hand, the divergence between destroyed and affected (1 to 9%), minor (10 to 25%), and major (26 to 50%) damage classes was relatively lower across all variables (Fig. 1). This explicit contrast provides a robust quantitative basis for assessing the contributions of variables to wildfire-induced building damage, as detailed in the following sections.

### Variable collinearity and principal components analysis

**Fig. 2.**
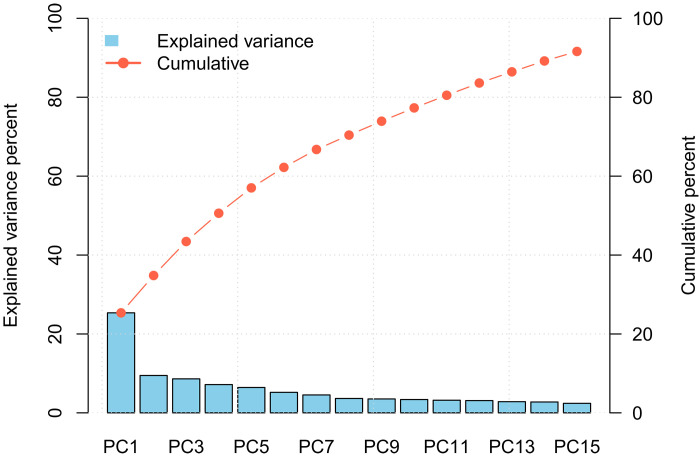
Variance explained by the first 15 PCs. The percentage of variance explained by the first 15 PCs and their cumulative variance.

**Fig. 3.**
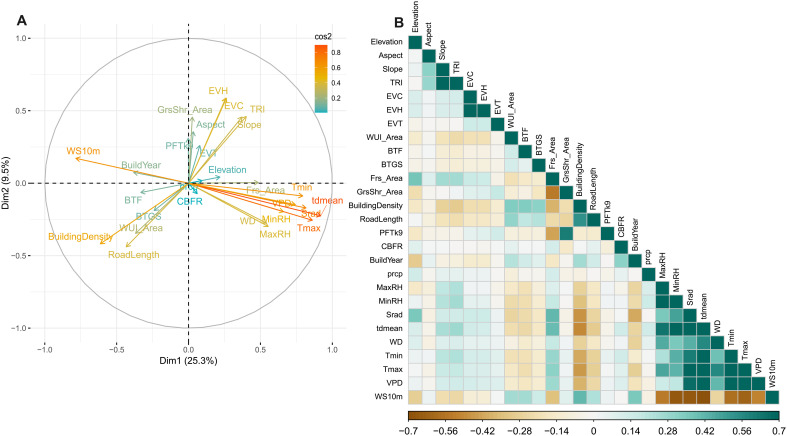
PCA variable contributions and intervariable Pearson correlation. (**A**) PCA cos^2^ showing variable contributions to PC1 (weather: tdmean, Srad) and PC2 (vegetation or terrain: EVC, Slope). Variables near the periphery are strongly represented. (**B**) Pearson correlation matrix of the variables, where green indicates a positive correlation and brown indicates a negative correlation.

The selected variables are broadly categorized into topographic, ecosystem, human footprint, building flammability, and weather variables to explain wildfire building damages. To examine the linear and nonlinear association, collinearity, and mutual overlapping among the variables, the study used the correlation coefficient, mutual information (MI), and principal components analysis (PCA). PCA identified patterns in the dataset, with the first five principal components (PCs) accounting for 57% of the total variance (Fig. 2 and table S2). PC1 alone accounted for 25.3% of the variance, strongly linked to weather variables, such as mean dew point temperature (tdmean, ${\text{cos}}^{2}=0.85$), solar radiation (Srad, ${\text{cos}}^{2}=0.67$), and maximum temperature (Tmax, ${\text{cos}}^{2}=0.74$), followed by vapor pressure deficit and wind speed (VPD and WS10m), emphasizing the importance of the thermal environment. PC2 explained 9.5% of the variance and was dominated by vegetation structure, with high loadings from existing vegetation cover (EVC; ${\text{cos}}^{2}=0.34$) and existing vegetation height (EVH; ${\text{cos}}^{2}=0.34$). In addition, terrain features such as slope and TRI $\left({\text{cos}}^{2}=0.21\right)$ differentiate rugged terrain from flatter terrain. PC3 (8.6% variance) was mostly influenced by land cover types, highlighting a gradient from forest (${\text{cos}}^{2}=0.45$) to grass/shrub areas (GrsShr_Area; ${\text{cos}}^{2}=0.53$) (Figs. 2 and 3). The correlation matrix reveals strong collinearity among several variables; notably, tdmean, Tmin, Tmax, and VPD are highly intercorrelated r≥0.70, suggesting redundancy; similarly, EVC and EVH (r=0.70) and Slope and TRI (r=0.70) also show significant overlap, indicating that dimensionality reduction or variable selection is warranted to avoid multicollinearity (Fig. 2). The correlation (Fig. 3B) and MI analysis exhibited the highest overlap in the group of weather variables (MI>1) (fig. S3).

The PCA and correlation analysis facilitated the minimization of the redundancy by eliminating variables with high collinearity (r>0.70) or overlapping PCA contributions $\left({\text{cos}}^{2}>0.7\right)$. From the correlated pairs, we retained Tmax (over Tmin), EVH (over EVC), and MaxRH (over MinRH) for their stronger climatic representation while discarding GrsShr_Area (redundant with ${\text{PFT}}_{k9}$) and BTGS (low contribution).

### Factors contributing to building damage

**Fig. 4.**
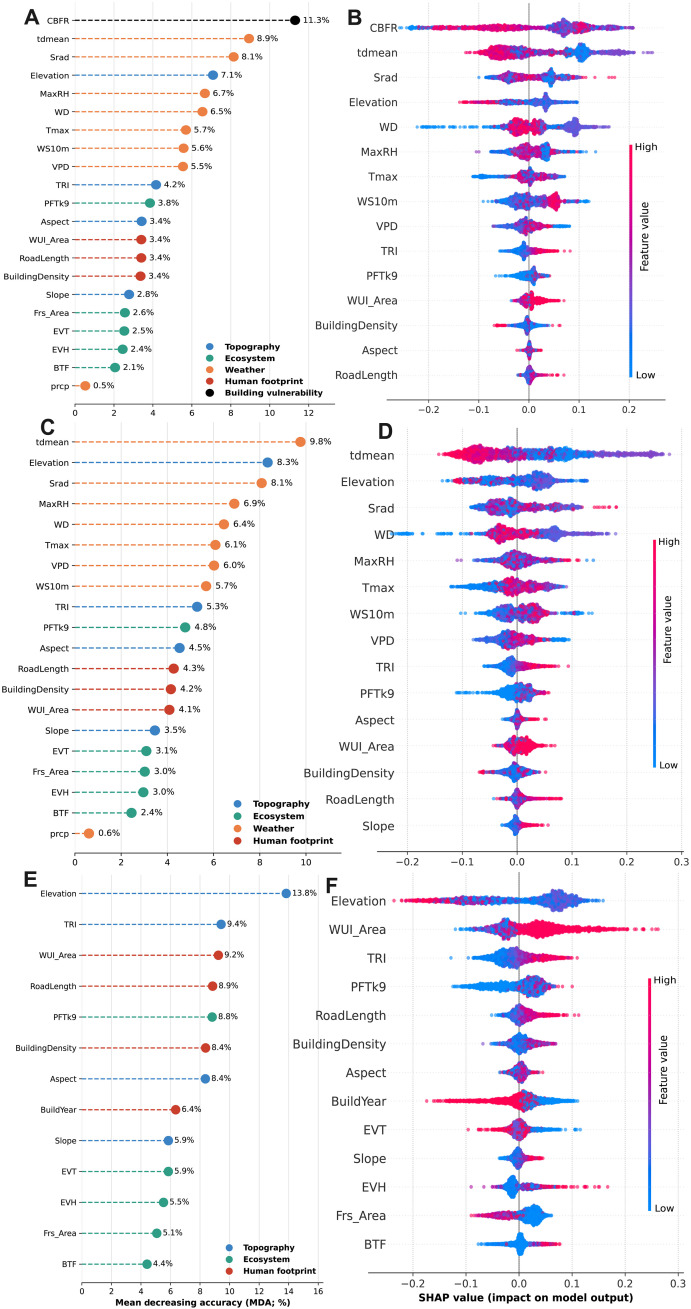
Variable importance in wildfire-induced building damage models. Variable importance in wildfire-induced building damage prediction using RF-based MDA and SHAP across three models: (**A** and **B**) comprehensive, (**C** and **D**) enviro-weather hybrid, and (**E** and **F**) environmental exposure model (SHAP showed 15 highest contributing variables).

The study reveals that building damage from wildfires is driven by complex interactions among environmental setup, building vulnerability, human activities, and weather conditions. The contributions of the selected variables from these groups are quantified using three different approaches (namely, the comprehensive model, the enviro-weather hybrid model, and the environmental exposure model, as discussed in Materials and Methods) to explain building damage [0, 1], using both mean decrease in accuracy (MDA) of random forest (RF) and SHapley Additive exPlanations (SHAP). The comprehensive model highlights differing perspectives on feature contributions, like the building vulnerability [Composite Building Flammability Rating (CBFR)], which is the most critical, contributing 11.3% to explain building damage, followed by daily mean dew point temperature (tdmean; 8.9%), solar radiation (Srad; 8.1%), elevation (7.1%), maximum relative humidity (MaxRH; 6.7), and wind direction at 10 m (WD10m; 7.2%) (Fig. 4A). The CBFR remains the highest contributor (21.40%) in both approaches; SHAP showed a mixed influence of CBFR in predicting building damage. The tdmean recorded the second most influential factor, contributing 16.20% on average to individual predictions with an inverse relationship: lower tdmean (drier air) greatly increases predicted building damage, while higher values decrease it (Fig. 4B and table S3). In addition, meteorological factors such as Srad (9.34%) and WS10m (6.90%) show significant contributions, often increasing damage under higher values. Terrain features (elevation and TRI), building density, and road length also emerge as moderate but consistent contributors. The enviro-weather hybrid model identifies tdmean (9.80%), elevation (8.3%), and Srad (8.10%) as the most influential predictors of building damage, as demonstrated by RF MDA (Fig. 4C) and SHAP (table S3). SHAP further indicates that lower tdmean (drier air) and higher wind speeds from the north-east and east significantly increase the likelihood of damage. Moderate elevation and higher exposure of the fine fuel (${\text{PFT}}_{k9}$) also contributed significantly (Fig. 4D).

The environmental exposure model, which focused exclusively on static variables, identifies elevation (13.8%), TRI (9.4%), WUI area (9.2%), road length (8.9%), and ${\text{PFT}}_{k9}$ (8.8%) as the most influential contributors (Fig. 4E). SHAP further reveals that moderate elevation and a greater proportion of WUI area are generally associated with increased predicted damage (Fig. 4F). In addition, the higher building density and older buildings (as older buildings tend to be more vulnerable) make a reasonable contribution to defining damage. While the dynamic fire-weather variables are excluded in this model, the results highlight how static exposure elements alone can significantly shape building damage outcomes (Fig. 4, E and F, and table S3).

### Causal influence of variables in wildfire building damage

We estimated individual causal influence of environmental, human footprint, meteorological, and building vulnerability using CausalForestDML, yielding both average treatment effects (ATEs) and spatially heterogeneous conditional average treatment effects (CATEs). ATEs (Table 2) reveal that a one-percentile increase in VPD increases damage probability by 2.0% (ATE = 0.020), representing the strongest causal effect among all variables. Tmax and WS10m exhibited moderate positive effects (ATEs = 0.008 and 0.0024, respectively), while mean burn temperature (tdmean) showed a small protective effect (ATE = −0.004). These results indicate that atmospheric aridity, ambient temperature, and near-surface wind speed exert strong causal influences on wildfire building damage probability.

**Table 2.** CATEs on wildfire building damage. CATEs from selected individual treatments, indicating the influence on wildfire building damage.

Variable	CATEs
CBFR	0.0001
tdmean	−0.0040
WS	0.0024
Elevation	0.0015
BuildingDensity	−0.0011
WD	−0.0022
WUI_Area	0.0001
Slope	0.0006
VPD	0.0200
RoadLength	0.0030
${\text{PFT}}_{k9}$	0.0004
Tmax	0.0075

**Fig. 5.**
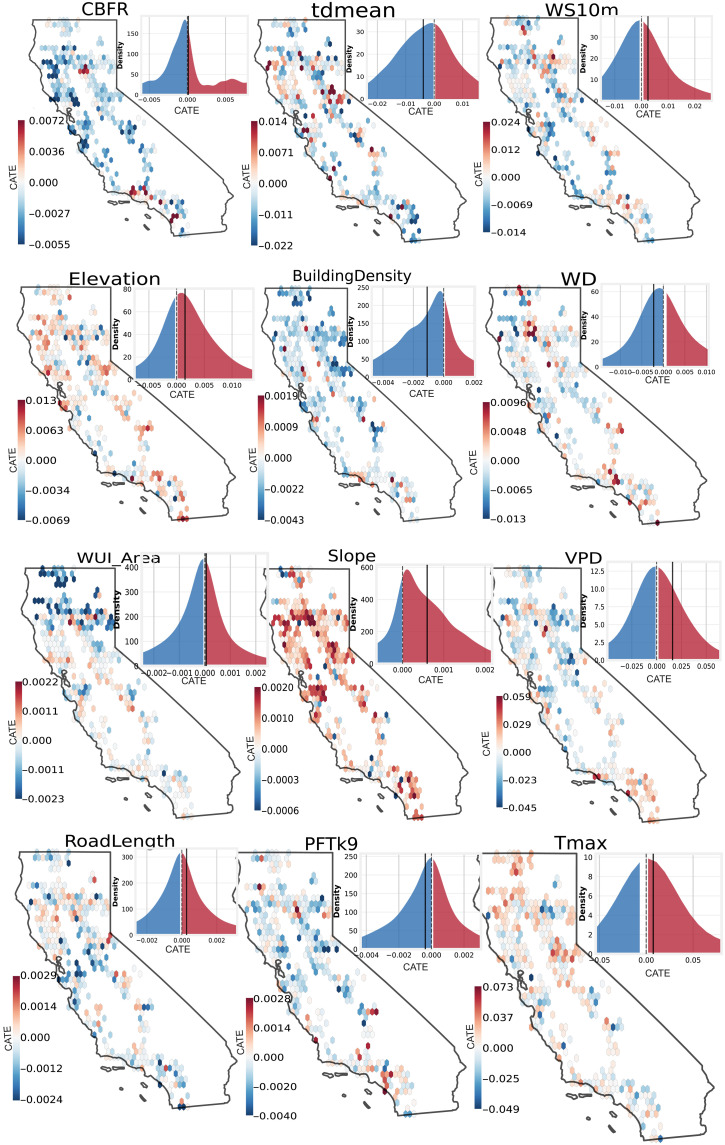
Spatial distribution of CATEs on wildfire-induced building damage. The CATEs quantify heterogeneous treatment effects on wildfire-induced building damage at the individual observation level. Each CATE value represents the estimated change in the probability of building damage per unit increase in the treatment variable. The hexagonal bins on the map denote the mean CATE within each geographic region, revealing spatial patterns in treatment effects for each treatment. The corresponding density plot illustrates the frequency density of CATEs across the study area, with positive values indicating an increased risk of damage and negative values indicating a decreased risk. The black vertical line on the density curve denotes the mean CATE.

Figure 5 presents spatial CATE maps alongside density distributions for each treatment, revealing substantial effect heterogeneity across California’s diverse wildfire landscapes. Meteorological variables exhibited the strongest spatial heterogeneity (CATEs). Tmax exhibited the largest CATE range (−0.049 to +0.073), with predominantly positive effects in northern and interior valleys. VPD showed the second-largest range (CATE = −0.045 to 0.059) with a spatially distinct pattern of strong positive effects in northern-central mountainous regions, and south coastal regions. The tdmean exhibited predominantly negative CATEs (−0.023 to 0.015), indicating protective effects from atmospheric moisture. Topographic variables showed moderate heterogeneity, elevation (−0.010 to 0.015), and slope (−0.05 to 0.002). Built environment variables building density (−0.0043 to 0.002) and road density (−0.0024 to 0.0029) displayed smaller, context dependent effects. The CATE distributions (Fig. 5) confirm that atmospheric variables demonstrate the largest magnitude and geographic differentiation, underscoring the importance of climate zone–specific mitigation strategies.

### Model performance and validation

The wildfire-induced building damage or no-damage [1, 0] has been modeled as a function of a set of variables using the RF binary classifier in three different approaches. The first approach, the comprehensive model, demonstrated a promising performance, achieving an overall accuracy (OA) of 0.88 and a weighted average of precision, recall, and F1 score of 0.88. This was followed by the enviro-weather hybrid and environmental exposure models, with overall accuracies of 0.81 and 0.74, respectively (Table 3). The confusion matrices of all the tests are presented in table S4. The receiver operating characteristic and area under the curve (AUC) values indicate strong class separation (AUC = 0.95 to 0.81), with minor variations reflecting differences in the feature sets (fig. S6).

**Table 3.** Classification performance. Accuracy matrix of RF classifiers of three different models.

Model	Class	Precision	Recall	F1 score
Comprehensive model	No-damage: 0	0.80	0.92	0.86
	Damage: 1	0.94	0.84	0.89
	Overall accuracy		0.88	
	Macro avg	0.87	0.88	0.87
	Weighted avg	0.88	0.88	0.88
Enviro-weather hybrid model	No-damage: 0	0.72	0.86	0.79
	Damage: 1	0.89	0.77	0.83
	Overall accuracy		0.81	
	Macro avg	0.81	0.82	0.81
	Weighted avg	0.82	0.81	0.81
Environmental exposure model	No-damage: 0	0.68	0.70	0.69
	Damage: 1	0.79	0.77	0.78
	Overall accuracy		0.74	
	Macro avg	0.73	0.74	0.73
	Weighted avg	0.74	0.74	0.74

To ensure model performance was not artificially inflated by local spatial autocorrelation, we first evaluated predictive skill under a strict 200-m spatial exclusion constraint. Under this rigorous 10-fold cross-validation (CV), the comprehensive model maintained a robust OA of 0.88% (± 0.004), significantly outperforming both the enviro-weather hybrid (0.82% ± 0.006) and environmental exposure (75.0% ± 0.006) models (table S7).

**Fig. 6.**
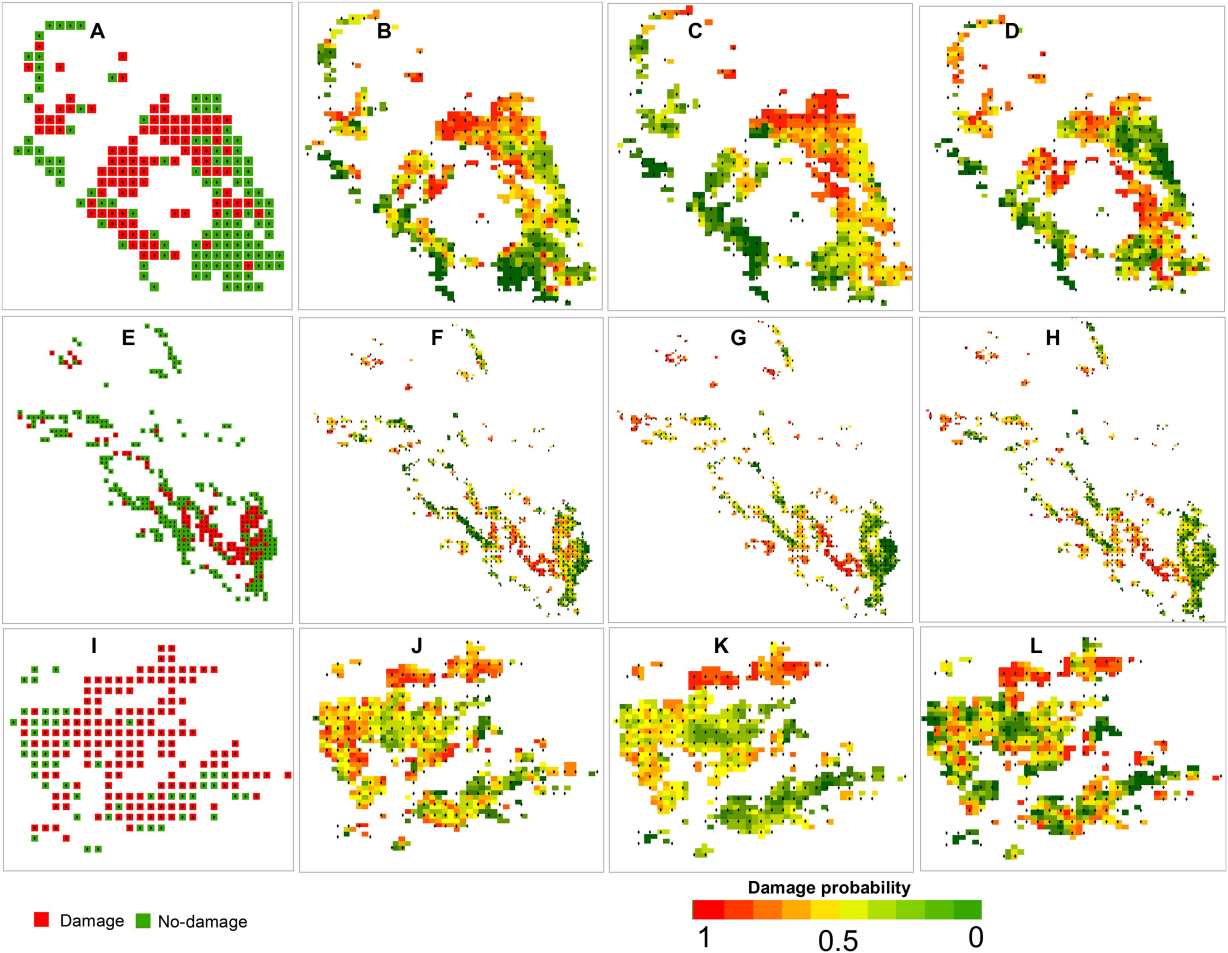
Observed and predicted building damage across major wildfire events. Building damage observations and predicted damage probabilities (ranging from 0 to 1) in 100 m across three major wildfire events: (**A**) CZU Lightning Complex Fire (2020), (**E**) Hennessey Fire (2020), and (**I**) Bear Fire (2024). Predicted damage probabilities are derived from the comprehensive model (**B**, **F**, and **J**), the enviro-weather hybrid model (**C**, **G**, and **K**), and the environmental exposure model (**D**, **H**, and **L**).

Furthermore, to assess event-level stability and out-of-sample predictive capability, the models were tested on three major, entirely withheld wildfires: the CZU Lightning Complex Fire (2020), the Hennessey Fire (2020), and the Bear Fire (2024) (Fig. 6 and table S5). The highest OA consistent with the above 10-fold CV, the comprehensive model scored the highest predictive performance across all three distinct fire events (OA = 0.76 to 0.82), whereas the environmental exposure model was the lowest (0.50 to 0.65).

**Fig. 7.**
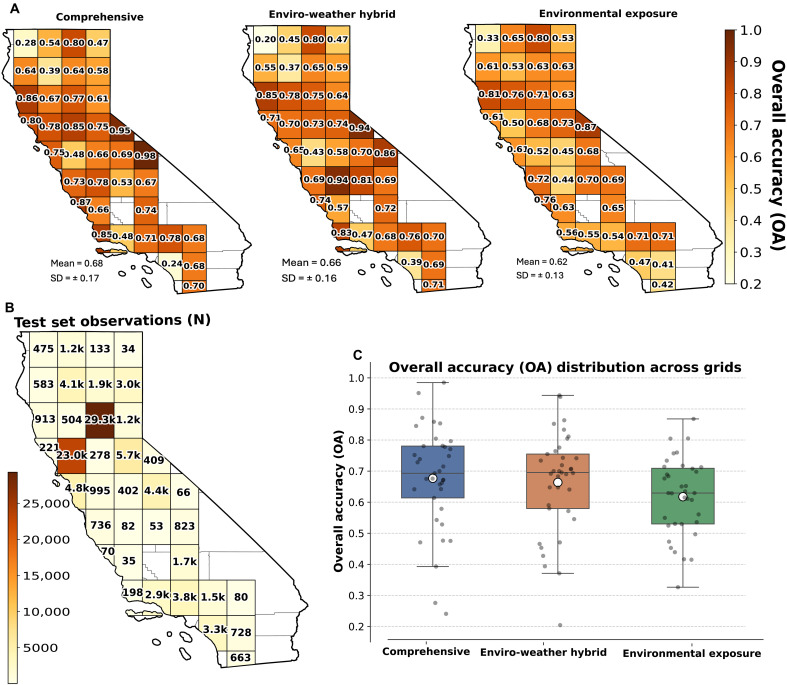
Spatial LOGO CV. (**A**) OA for each 1° by 1° geographic grid cell, where models were trained on all grids except the held-out test grid; (**B**) number of test observations (*N*) within each grid cell, and (**C**) boxplots showing the distribution of OA scores across grids, where the white dot represents the mean.

Last, we tested the models’ regional generalizability using a 1° by 1° spatial blocking approach [Leave-One-Grid-Out (LOGO)] CV (Fig. 7). While accuracy substantially varies across the grids, the comprehensive model consistently exhibited the highest spatial transferability, with an average OA of 0.68 (SD = ±0.17; Fig. 7, A and C). Notably, instances of reduced accuracy across grids were predominantly attributed to diverse ecological settings, further compounded by insufficient test observations (Fig. 7B).

**Fig. 8.**
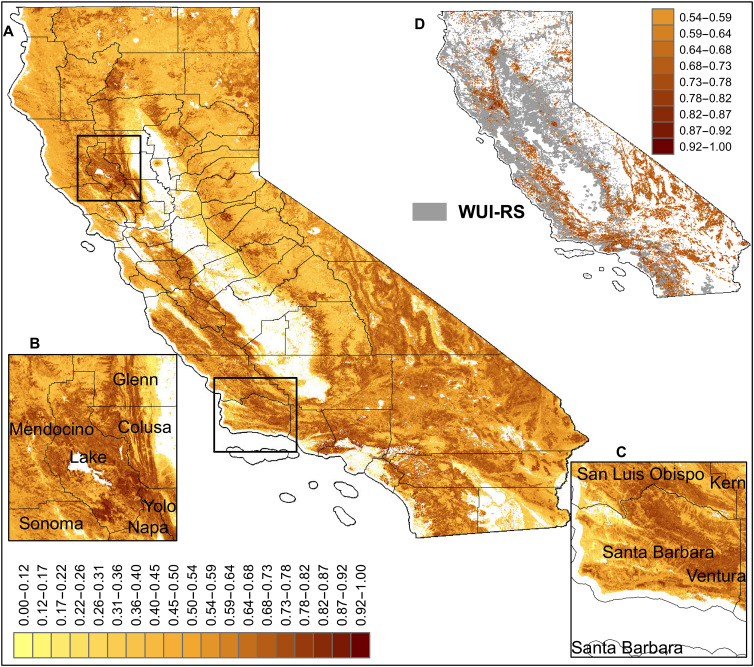
WBDR across the study region. WBDR [0–1] derived from the environmental-exposure model. (**A**) Spatial distribution of WBDR across the study area; (**B**) zoomed-in view around Lake County; (**C**) zoomed-in view around Santa Barbara County; and (**D**) spatial integration of WUI [Li *et al.* (*52*)] with WBDR values exceeding the third quartile.

The validation framework demonstrated that building vulnerability and real-time fire-weather conditions are critical drivers of building damage risk. However, despite these performance trade-offs, the static environmental model retains predictive skill to be practically valuable. Consequently, the granular (100-m) wildfire building damage risk (WBDR) layer (Fig. 8, A to C) remains pragmatically useful for prioritizing fuel management from the building’s surroundings to community scales, particularly when real-time comprehensive data are unavailable.

### Developing a WBDR map

Maps of WUI have been regarded as one of the most reliable tools for defining areas at risk of infrastructure damage from wildfire. However, wildfire risk within the WUI is highly heterogeneous and dynamic, influenced by context-specific factors such as weather, terrain, fuel characteristics, and the presence or absence of mitigation measures. In contrast, the WBDR map introduced in this work (discussed in Materials and Methods) could offer a spatial gradient of damage risk in and around the WUI. A spatial integration of higher–damage risk zones, specifically the third quintile of WBDR (>0.55), along with the latest remote sensing–based WUI by Li *et al*. (*38*), presents a greater opportunity to identify areas with comparatively higher risk of damage (Fig. 8D). By combining the granular WBDR layer with an integrated WUI approach, our multiscalar analysis yields a nuanced spatial gradient of risk that can inform targeted mitigation strategies. However, the Damage Inspection (DINS) postfire investigation covered 22 fires in California between 2013 and 2024, and they recorded no damage records from 2018 (*11*), which may not reflect the complete extent of observations of fire infrastructure damage in the area. In addition, integrating rapid postfire damage detection from EO (*32*) and DINS data could significantly enhance the accuracy and depth of postfire information.

## DISCUSSION

This study, grounded in DINS postfire observations, gridded EO data, reanalysis of weather, and multiple statistical and causal modeling frameworks, demonstrated that wildfire-induced building damage results from the intricate interaction of static topographic setup, dynamic fire-weather conditions, vegetation, building vulnerability, and human footprint. Over a decade of postfire investigation from CAL FIRE (2013 to 2024) reveals that 86% of damaged buildings are located within the WUI, with 53,879 structures destroyed. This concentration of destruction in WUI underscores its inherent vulnerability, stemming from the juxtaposition of flammable vegetation and densely populated human infrastructure. Among all variables examined, atmospheric aridity (VPD; ATE = 0.020), maximum temperature (Tmax; ATE = 0.008), building flammability (CBFR), and mean dew point temperature (tdmean; ATE = −0.004) emerge as the most dominant causal influences in building damage probability. Spatial heterogeneity analysis reveals that Tmax effects are strongest in interior valleys (CATE: −0.049 to +0.073), while VPD exhibits the strongest influence in northern central mountainous regions (CATE: −0.045 to +0.059), indicating that temperature-driven and atmospheric aridity-driven damage mechanisms operate through geographically distinct pathways across California’s physio-climatic gradients. The protective effect of tdmean confirms that higher atmospheric moisture consistently reduces damage probability by increasing fuel moisture content, consistent with evidence that reduced humidity amplifies ignition potential (*39*).

Our analysis indicates that fence materials carry the highest influence (16%) on building vulnerability, followed by exterior siding (15%), build year (14%), roof materials (13%), and eaves type (10%) (Table 4). Note that Papathoma-Köhle *et al*. (*7*) found that the roof material (33%) and structural type (23%) stand out the highest weightage in defining the vulnerability of buildings to wildfires, based on observations from California and Greece. The space between buildings, exterior siding, and building age showed the strongest influence on damage risk (*37*). Wood siding and decks are major ignition sources during wildfires due to their high susceptibility to ember attack (*40*). The topographic variables suggest that mid-elevation zones (522.00 m, ±370.32m) with moderate slopes ($5^{\circ },\pm 4.76^{\circ }$) and less rugged terrain (TRI: 7.21,±6.9) are more susceptible to damage, likely because of their influence on fire spread and intensity through enhanced fuel connectivity and wind channeling. The fine fuel (shrubs/grasses) domination around the buildings and low-to-mid canopy trees, such as California Annual Grassland, Mesic Chaparral, and Mixed Oak Woodland, further exacerbates damage risks because of their high flammability and rapid combustibility.

**Table 4.** Feature weightage in building flammability assessment. Weightage of building structure and composition features in building flammability rating, retrieved by the Boruta algorithm.

Feature	Weight
Fence	0.16
Exterior	0.15
Build year	0.14
Roof materials	0.13
Eaves	0.10
Building type	0.09
Deck porch	0.06
Patio cover	0.05
Vent screen	0.05
Windowpane	0.05

SHAP and RF-based modeling approaches offer deeper insights into the relative influence of each variable that drives wildfire damage. The building flammability (CBFR) stands out as the most significant factor (11.3% contribution in the comprehensive model), highlighting the critical importance of structural fire resistance, as well as defensible space regulations and building code enforcement. (*27*, *28*) also reported that structural features have the highest predictive capacity to determine whether a building will survive or be destroyed, and that enclosed eaves, vent screens, and multipane windows help protect against embers and radiant heat. The atmospheric dryness, quantified by tdmean, plays a critical role; lower tdmean values significantly increase the probability of building damage, indicating that drier air conditions escalate the speed of fire spread and combustion potential. The substantial contributions of higher wind speed from the northeast and east and near-surface temperature further enhance fire line intensity, spotting potential, and directional fire behavior (*41*); a similar fire-weather pattern has been observed in large-fire events over the west coast (*42*, *43*).

The spatially heterogeneous causal effects (CATEs) identified across the study area suggest implications for targeted wildfire risk mitigation strategies based on environmental and anthropogenic gradients. The VPD and Tmax exhibit distinct geographic patterns of influence, suggesting that fire weather-based early warning systems should be calibrated to regional climate zones. In hot interior valleys where Tmax drives the strongest effects, pre-positioning firefighting resources and issuing evacuation warnings during extreme heat events would be most effective. Conversely, in mountainous forest zones where VPD and tdmean effect dominate, monitoring atmospheric aridity thresholds and live fuel moisture stress should guide proactive prescribed fire and mechanical thinning operations during windows of lower fire risk (*44*, *45*). The strong influence of CBFR on building damage indicates that structural hardening, including replacing wood fencing, upgrading siding to noncombustible materials, retrofitting eaves, and installing ember-resistant vents, can substantially reduce damage probability. California’s SB 504 (2024) (*46*, *47*), mandating 5-foot (152.40-cm) ember-resistant zones adjacent to structures, aligns with our finding that fine fuel exposure (${\text{PFT}}_{k9}$) significantly increases risk.

The building damage probability map, WBDR (Fig. 8), combined with WUI delineations, facilitates the spatial identification of structural risk zones. It complements and extends existing wildfire risk mapping products. Unlike traditional WUI delineations (*3*, *38*), which classify areas as interface or intermix on the basis of housing footprints and vegetation thresholds, WBDR provides a continuous gradient of damage probability (0 to 1) at 100-m resolution, capturing within WUI heterogeneity driven by topographic and ecosystem exposure. This finer-scale risk stratification enables more targeted allocation of mitigation resources than that provided by binary WUI classifications alone. Local jurisdictions and homeowners could use the WBDR map to prioritize enforcement of defensible space inspections in high-risk zones (WBDR > 0.55), where the return on investment for fuel reduction and structural hardening is highest.

### Limitations

While our integrated RF, SHAP, and causal learning framework provides meaningful insights into wildfire-induced building damage, certain limitations should be noted. The reliability of predictions depends on the quality of input data, including EO derived vegetation layers (LANDFIRE-based EVT, EVH, and EVC) and National Land Cover Database (NLCD) land cover, which contain classification errors that may propagate through the models. Sensitivity analysis (fig. S4) indicates that key variables CBFR, BuildingDensity, and WUI_Area for the comprehensive model; Elevation, tdmean, and WS10m for the enviro-weather hybrid model; and WUI_Area, BuildingDensity, and ${\text{PFT}}_{k9}$ for the environmental exposure model exert the greatest influence on model predictions, highlighting that uncertainties in these inputs could meaningfully affect predictive accuracy. The DINS database, with 22 fires (2013 to 2024), does not capture all structure losses statewide, particularly for smaller fires or events outside CAL FIRE’s jurisdiction. In addition, DINS recorded no damage observations in 2018 (*11*), creating a temporal data gap. Integrating DINS with satellite-based rapid damage detection (*32*) and crowd-sourced postfire damage reports could enhance spatial coverage and temporal continuity. We modeled building damage at a spatial resolution of 100 m to capture neighborhood- and building-level damage risk and its influencing variables. At this scale, multiple structures can fall within the same grid cell. Such spatial proximity can lead to nonindependent observations and model bias, as nearby buildings are likely to experience similar environmental conditions, fire behavior, and damage outcomes. In addition, while our 200-m dead-zone and spatial blocking CV (LOGO) approaches mitigated local and regional spatial leakage, they highlighted that predictive transferability remains inherently challenged by California’s extreme geographical diversity. Accuracy naturally degraded when transferring the models to entirely unseen, ecologically distinct domains that lack sufficient historical observation data. Future work should integrate social vulnerability dimensions, including household income, renter occupancy, aging populations, and evacuation accessibility, with WBDR and physics-based fire behavior components to pinpoint communities confronting intersecting wildfire and socioeconomic challenges.

In conclusion, this study presents a comprehensive, physically interpretable data-driven framework that integrates structural, environmental, and meteorological variables to support causal inference and predict wildfire-induced building damage across California. This study identifies building flammability, fine-fuel exposure, fire weather (dew point temperature and wind speed), and a fraction of WUI around buildings as the dominant drivers of wildfire-induced building damage, leveraging explainable ML and multisource geospatial data. Around 86% of damaged structures occur in WUI zones, where the interplay of flammable vegetation (e.g., California Annual Grassland), building materials, and topographic factors (mid-elevation slopes) amplifies risk. The wildfire-induced building damage is driven not by isolated factors but by the confluence of extreme fire weather (atmospheric aridity, temperature, and windspeed), flammable landscapes (fine fuel dominance, mid-elevation topography), structural vulnerability (CBFR components: fencing, siding, and eaves), and spatial exposure patterns (WUI configuration and building density). A heterogeneous causal influence of variables on building damage across the study area suggested implications for a targeted wildfire risk mitigation strategy based on environmental and anthropogenic gradients.

By integrating static environmental and dynamic fire-weather variables into a granular WBDR layer, we provide a spatially explicit tool for prioritizing mitigation measures (e.g., fuel reduction and fire-hardened structures). Moving forward, operational tools can support localized, proactive strategies to reduce future wildfire losses, emphasizing the need for integrative approaches to protect communities at the WUI. Future efforts should expand structural datasets, physical processes modeling, and a data-driven framework to enhance predictive capacity. Still, our findings already advocate for integrated strategies combining building codes, land-use planning, and vegetation management to reduce vulnerability in high-risk zones.

## MATERIALS AND METHODS

### Study area

The study was carried out over the West Coast of the US, California (fig. S1), including several different eco-regions, such as the Mediterranean California, the Northwestern wooded mountains, the North American deserts, and the Marine West Coast Forests. The climate of California varies significantly across different eco-regions. The diverse geography, including the Sierra Nevada and other high areas with steep slopes and alpine zones, contributes to complex fire behavior by altering fuel types, moisture content, and wind patterns (*48*). In the northwest, thick coniferous forests prevail, whereas in the south and interior regions, vast chaparrals and grasslands dominate, creating a higher fire risk in these areas (*49*, *50*). In addition, California has a high population and construction density, especially in coastal cities like Los Angeles and San Francisco, where development spills into WUI regions that are prone to infrastructural damage from fire (*8*, *51*).

### Wildfire building DINS data and WUI

The study investigates the insides of the building damaged from wildland fires using the CAL FIRE DINS data. The advantages of DINS data include rich, geo-coded information on the scale of damages, the building’s structural information (age of the building, types), and the composition of materials (roof, vents, eaves, exterior walls, deck, and windows) (*11*). The study ingested the inspection data of 100,230 observations between 2013 and 2024 over California (fig. S1). The damage scale is divided into five major categorical groups, i.e., no damage, affected (1 to 9%), minor (10 to 25%), major (26 to 50%), and destroyed (>50%), on the basis of the intensity of the damage. The study also used the latest remote sensing–derived WUI (WUI-RS) data from (*52*) to incorporate wildland-human infrastructure transition gradients into the building damage study.

### Building density and road

Building density is an exposure component in the wildfire damage process; the high-resolution building footprint from (*53*) has been incorporated to inform it. However, the road network serves as a barrier or break component in the process of fire spread, and it defines the accessibility for firefighting and evacuation. The study used the US Census Bureau and TIGER line shapefiles, and the road density was computed for every 100-m grid (*54*).

### Topographic and ecosystem descriptors

A landscape’s topography and ecosystem components form the primary boundary layer of wildfire, shaping the geography and fuel bed that determine fire ignition and spread. The study used the essential topographic (i.e., elevation, slope, aspect, and roughness) and ecosystem [EVC, type (EVT), and height (EVH)] from LANDFIRE (*55*) and NLCD (*56*). This could explain the physiographic and ecosystem components around the buildings. The forests and grass-shrub areas, as well as their relative presence around the buildings, were retrieved using the 30-m resolution NLCD land-use land cover for each year from 2013 to 2024. The study found that the proportion of these PFTs within 1 km of buildings significantly controls wildfire building damage (*27*). To explore these associations, we compute a fuel ratio using fine (grassland and shrubland) and woody (forests) fuels to represent the proportional presence of each fuel type around the building, with a kernel size of 9 by 9 (${\text{PFT}}_{k9}$), which was calculated using the following (*1*)

$${\text{PFT}}_{k9}=\frac{n_{\text{FineFuel}}}{n_{\text{WoodyFuel}}}$$ (1)

where $n_{\text{FineFuel}}$ and $n_{\text{WoodyFuel}}$ represent the number of fine and woody fuel pixels (30-m resolution) within a 9 by 9 kernel, respectively. Among the tested kernel sizes ranging from 3 by 3 to 11 by 11, the 9 by 9 kernel exhibited the best performance in explaining building damage (fig. S7). The selection was based on the MDA metric obtained from the RF model. These selected variables, among others, have reasonable controls in the local wildland fire behavior vectors: flame length, fire line intensity, and rate of spread (*1*, *8*, *10*, *51*).

### Weather factors

Weather is the most dynamic component driving wildland fire behavior, encompassing factors such as temperatures, precipitation, wind, atmospheric humidity, and vapor pressure. These components interact with the geographic and ecosystem conditions of the landscape to shape fire dynamics (*57*). A combination of hot, dry, and windy conditions, along with supportive geographic conditions, produces a conducive wildfire ignition and spread, like the recent devastating Los Angeles fire (January 2025), with ∼40,687 acres burnt and 16,000 structural losses (*58*). The study used daily gridded weather datasets over the damaged dates from PRISM (Parameter-elevation Regressions on Independent Slopes Model) (*59*) and GRIDMET (*60*) at a spatial resolution of 4638.3 m.

### Building flammability rating

Building ignition from wildfires occurs through direct flame impingement, radiant heat, and ember exposure (*25*), yet a structure’s susceptibility is primarily determined by its specific type, structure, and materials (*2*, *12*, *28*). This study uses structural data from the DINS database, including building age, eaves, window structure, roof, and exterior materials, to assess these vulnerabilities. To quantify flammability, each factor was assigned an independent rating on a scale of 0 to 10, where 0 signifies noncombustibility and 10 indicates high flammability, derived from material classifications (*61*) and a comprehensive evaluation of relevant literature [table I in the study of Dossi *et al.* (*8*) and table S6].

To bridge the gap between these laboratory-tested material classifications and literature-based flammability ratings and real-world wildfire damages, we used the Boruta algorithm (*62*) as a calibration layer to extract weights reflecting the observed significance of each feature in characterizing building damages. We use a stratified random sample of 30% of observations, proportionally balanced across DINS damage classes (No Damage, Affected, Minor, Major, and Destroyed) for Boruta-based calibration of flammability ratings. The Boruta algorithm, a RF-based wrapper method, determines the relative importance of each structural attribute by comparing feature significance against randomized shadow features (*62*), yielding normalized calibration weights (Table 4). This design ensures that 70% of observations receive weights learned on completely independent data, substantially reducing circularity compared with using the full dataset for both calibration and prediction. This supervised feature engineering approach transforms independent structural and material flammability ratings into a physically grounded CBFR through an additive function, where each rating is multiplied by its respective Boruta-derived weight

$$\text{CBFR}=\sum_{i=1}^{10}w_{i}\times R_{i}$$ (2)

where $R_{i}$ above is the given flammability rating (0 to 10 scale) and $w_{i}$ is the Boruta-derived calibration weight (%) for structural attribute *i*. CBFR values were rescaled to a 0 to 10 range using min-max normalization. By calibrating literature-based flammability scores with field-observed wildfire behavior, the CBFR serves as an optimized, dimensionally reduced index that effectively reconciles laboratory-based material science with real-world damage patterns in the California WUI.

### Modeling framework to explain wildfire building damage

Wildfire-induced infrastructure damage is primarily guided by fire behavior (intensity, spread rate, and embers), building exposure, and resilience (*8*). The component of landscape ecology provides a valuable template for assessing both fire behaviors and their impacts (*63*). The study has used landscape ecological components, including environmental variables, human footprint, and weather conditions, to explain wildfire-caused building damage across California and to develop an environmentally based wildfire building damage potential matrix. We assume that building damage is influenced by both fire behavior and the building’s vulnerability; the environment, human footprint, and weather conditions shape fire behavior. To investigate and model the influence of the building’s surroundings, including fuel proportions and density of infrastructure, on the other hand, to maintain the granularity, we conducted the study with a spatial resolution of 100 m across California.

This study analyzed 100,230 postfire building inspection records from CALFIRE (2013 to 2024). Among these, 58.6% of the observations fell into the category of minor damage to destroy, while 40.8% had no damage. To evaluate the distribution of building damage classes across the static environmental factors, we used three statistical approaches: (i) probability density estimation to identify the distribution of the variables for each damage class, (ii) Kruskal-Wallis rank sum tests to check the null hypothesis, i.e., no significant variability of the above factors across different damage classes Eq. 3, and (iii) post hoc tests, Dunn’s with Bonferroni correction Eq. 4 to quantify the degree of pairwise divergence of destroyed and other damage classes (*64*). This nonparametric framework accommodates non-normal distributions while controlling family-wise error rates in multiple comparisons. The Kruskal-Wallis *H* statistic is calculated as follows

$$H=\frac{12}{N\left(N-1\right)}\sum_{i=1}^{k}\frac{R_{i}^{2}}{n_{i}}-3\left(N+1\right)$$ (3)

where *N* is the total number of observations across all groups, *k* is the number of groups, $n_{i}$ is the number of observations in group *i*, and $R_{i}$ is the sum of ranks in group *i*.

To perform post hoc pairwise comparisons, Dunn’s test is used with the following *z*-statistic

$$z=\frac{{\bar{R}}_{i}-{\bar{R}}_{j}}{\sqrt{\frac{N\left(N+1\right)}{12}\left(\frac{1}{n_{i}}+\frac{1}{n_{j}}\right)}}$$ (4)

where *z* is the Dunn’s test statistic for comparing groups, *i* and *j*, ${\bar{R}}_{i}$ and ${\bar{R}}_{j}$ are the average ranks of groups *i* and *j*, respectively, $n_{i}$ and $n_{j}$ are the number of observations in groups *i* and *j*, and *N* is the total number of observations.

The chosen static (topographic, ecosystem, and human footprint) and dynamic variables (weather) were examined using PCA (*65*), MI (*66*), and the Pearson correlation coefficient (*67*) to discern underlying associations and the direction of relationships among variables in a reduced-dimensional framework. This study adopted three distinct approaches to modeling and analyzing wildfire-induced building damage, assessing the contribution of various factors through different combinations.

1) Comprehensive model: Integrates both static variables (topography, ecosystem, human footprint, and building flammability) and dynamic variables (weather conditions) to explain the extent of building damage from wildfires.

2) Enviro-weather hybrid model: Focuses on static environmental and dynamic weather variables, excluding the building flammability rating (CBFR).

3) Environmental exposure model: Incorporates only environmental static components (including topography, ecosystem, and human footprint), excluding building-specific characteristics and dynamic weather influences.

The DINS building damage categories (fig. S1) were reclassified into two binary classes: damage and no damage. The damage class comprises affected (1 to 9%), minor (10 to 25%), major (26 to 50%), and destroyed (>50%). After the reclassification, 59% of the observations were damaged, while the remaining 41% were classified as no damage. To reduce the risk of model bias toward a particular class and to address the class imbalance between damaged and no-damage observations, we applied the synthetic minority oversampling technique on the training dataset only, which balances the classes without introducing additional information into the dataset (*68*). Using this balanced dataset, the RF (*69*) was used to develop the three models mentioned earlier to compare the relative influence of environmental factors, human footprint, building flammability, human presence, and weather dynamics on wildfire-induced building damage. A nested *k*-fold CV with the grid-searching approach (*70*) was used to identify robust hyperparameters for the RF models, followed by training on 70% of the data and testing on the remaining 30%. The models were evaluated using accuracy metrics, including F1 score, precision, recall, and OA. To assess the models’ overall consistency and rigorously mitigate spatial autocorrelation, we applied a 10-fold CV strategy. During each fold, a 200-m spatial “dead-zone” was enforced, systematically excluding any training point located within 200 m of the testing points. This approach prevents local spatial data leakage by ensuring the model is not trained and tested on adjacent, highly correlated samples.

We also evaluated the fire-specific performance of the models using distinct exposure datasets for three large fires (table S5). These three fires were selected to represent California’s WUI diversity in terms of geography, environmental conditions, and development patterns. They range from coastal forests (CZU Lightning Complex), interior oak woodlands (Hennessey), and montane conifer forests (Bear) (*41*, *52*, *55*), covering distinct fuel types, topographies, and WUI prevalent across the study area.

Last, to evaluate broad-scale regional transferability and mitigate spatial data leakage, we used a LOGO spatial CV approach. The study area was divided into 1° by 1° geographic grids. In each iteration, the models were trained on all available data except for one grid, which was entirely withheld to serve as an independent, geographically distinct test set. This spatial blocking approach ensures that the model’s predictive capabilities are evaluated on a completely unseen ecological region.

Input sensitivity was assessed by selecting the five most important variables from each model on the basis of the MDA of RF. Each variable was perturbed by ±10%, and the resulting changes in predicted probabilities were quantified and visualized using probability density curves (fig. S4).

In addition, SHAP was used to interpret the influence of individual variables in the RF model, providing richer local insights into feature importance. SHAP uses the Shapley interaction index from game theory to effectively capture local interaction effects (*71*, *72*). Integrating SHAP with RF disentangles the proportional contributions of selected factors and their variation across value ranges, revealing nonlinearities and interactions in wildfire-induced building damage that aggregate importance measures may overlook.

To ensure the scalability of the study, we used the environmental exposure model to obtain a WBDR layer, which predicts the likelihood of building damage at a spatial resolution of 100 m. Notably, the CBFR was excluded from this specific model, as detailed building-level structural information is not uniformly available across the study area. By excluding the CBFR, the WBDR serves as an independent assessment of how environmental and exposure factors alone drive damage likelihood. In addition, a comparative analysis was performed between WUI-RS (*38*) and WBDR to evaluate the distribution of wildfire building damage, and a new metric, Wildfire Building Damage Potential WUI (WBDP-WUI), was developed, integrating the higher WBDR regions with WUI-RS.

### Causal effects in wildfire building damage

This study quantifies the causal effects of environmental conditions, the human footprint, and building vulnerability on wildfire-induced building damage using a robust causal machine learning framework under conditional independence assumptions. Analogous to applications in medicine, where causal ML is used to estimate treatment effects under confounding (*34*), we apply this paradigm to wildfire risk modeling to assess how marginal changes in modifiable factors (e.g., meteorological conditions, landscape structure, or built-environment characteristics) causally influence damage probability while adjusting for correlated spatial and environmental drivers. Estimating heterogeneous treatment effects is particularly important in wildfire systems, where the effectiveness of interventions varies substantially across local topography, climate, and neighborhood context.

Each variable is treated as a continuous exposure, and causal effects are interpreted as the marginal change in building damage probability associated with an increase in that exposure, conditional on selected confounders. A similar approach has been used in studies (*73*, *74*) to explain environmental components following recent advances in causal ML (*34*, *75*).

For each treatment, we perform treatment-specific confounder selection, acknowledging that different exposures require different adjustment sets. Confounders are selected on the basis of a minimum association threshold of 10% (Spearman’s ρ>0.1) with both the treatment and the outcome, following established practices in observational causal analysis (*76*). The selected confounders for each treatment are listed in fig. S5.

We use double ML (DML) with CausalForestDML (*34*, *74*, *77*, *78*), which is well suited for continuous treatments and high-dimensional confounding. The CATE for individual *i* with covariates $X_{i}$ is estimated as

$$\tau \left(X_{i}\right)=E\left[Y_{i}\left(1\right)-Y_{i}\left(0\right)|X_{i}=x\right]$$ (5)

where $Y_{i}\left(1\right)$ and $Y_{i}\left(0\right)$ represent potential outcomes under treatment and control, respectively, and $X_{i}$ denotes the covariate profile. The ATE is obtained by averaging CATEs across all observations.

Last, we present spatial maps of CATEs for individual treatments, illustrating how the magnitude and direction of causal effects vary across environmental and human exposure components. These spatial patterns support the interpretation of the estimated effects as true heterogeneity driven by local context rather than spurious associations.

## Supplementary Materials

### This PDF file includes:

Figs. S1 to S7 Tables S1 to S7 References
