# Supplementary material for: Explaining building damage from wildfires in California
Source: Sci Adv. 2026 Jul 24;12(30):eaed4197. doi: 10.1126/sciadv.aed4197 (PMC13398487; doi:10.1126/sciadv.aed4197)
Supplement: Supplementary file 1 — Figs. S1 to S7 Tables S1 to S7 References [file sciadv.aed4197_sm.pdf]

Science Advances
AAAS

Supplementary Materials for

Explaining building damage from wildfires in California

Somnath Bar *et al.*

Corresponding author: Somnath Bar, somnatb@uci.edu

Sci. Adv. **12**, eaed4197 (2026)
DOI: 10.1126/sciadv.aed4197

This PDF file includes:

Figs. S1 to S7
Tables S1 to S7
References

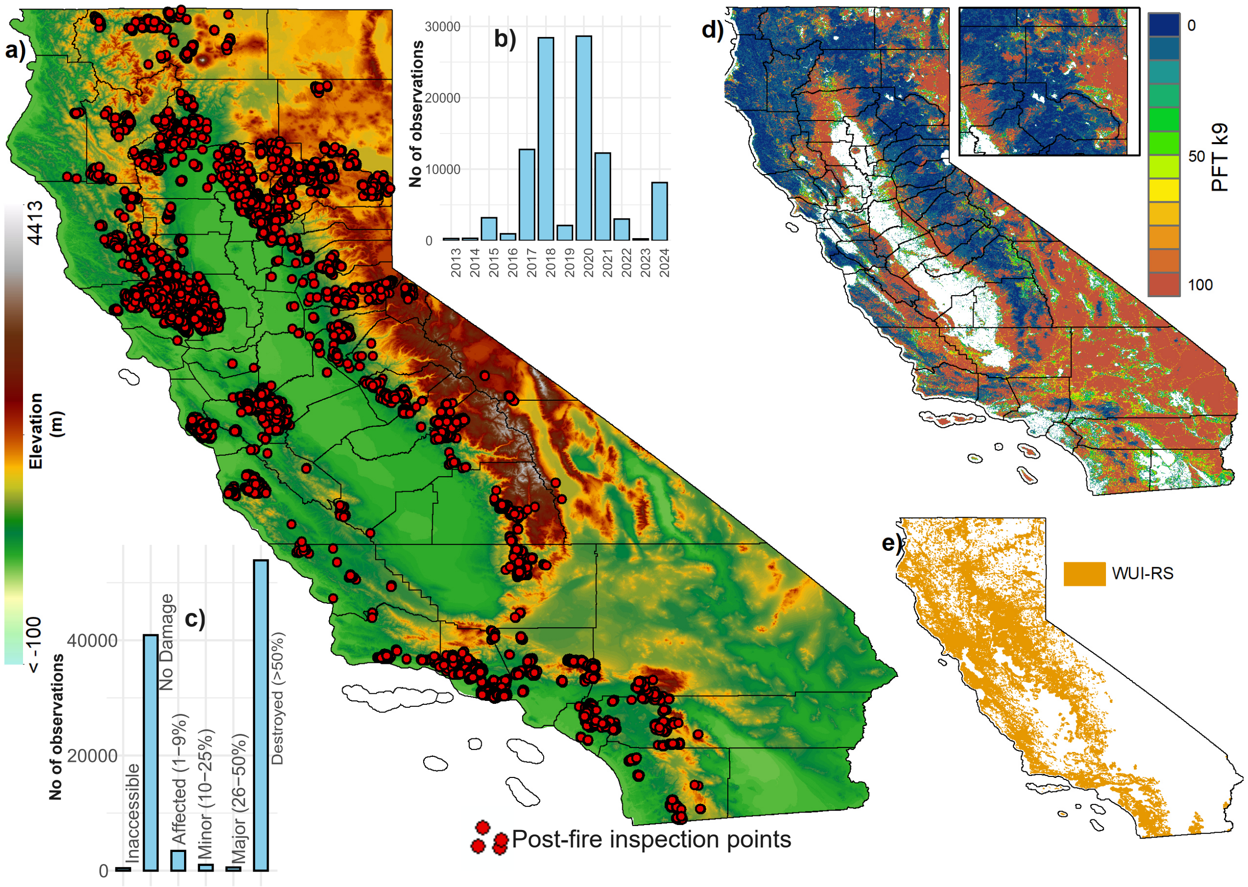

Figure S1: a) Topographic elevation of the study area, California; b) yearly observations of post-fire building damage points from 2013-2024; c) observations in different damage classes by DINS, CAL FIRE; d) proportions of plant functional types at a kernel size of 9×9 [values close to 100: higher dominance of grass/shrubs, values close to 1: higher dominance of trees]; and e) spatial extent of WUI by Li et al. (*39*).

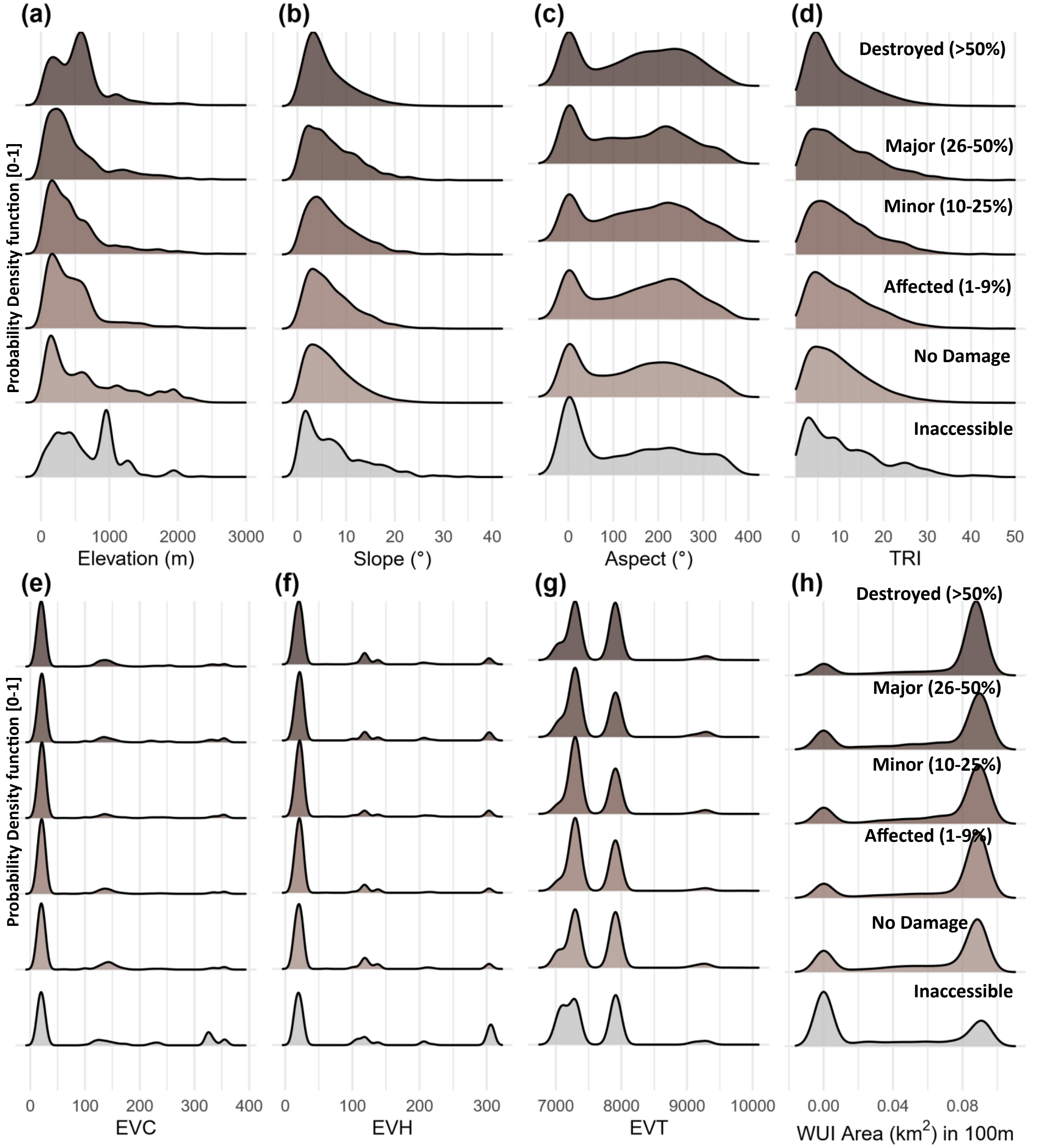
(a)
(b)
(c)
(d)
Destroyed (>50%)
Major (26-50%)
Minor (10-25%)
Affected (1-9%)
No Damage
Inaccessible
Probability Density function [0-1]
Elevation (m)
Slope (°)
Aspect (°)
TRI
(e)
(f)
(g)
(h)
EVC
EVH
EVT
WUI Area (km2) in 100m

Figure S2

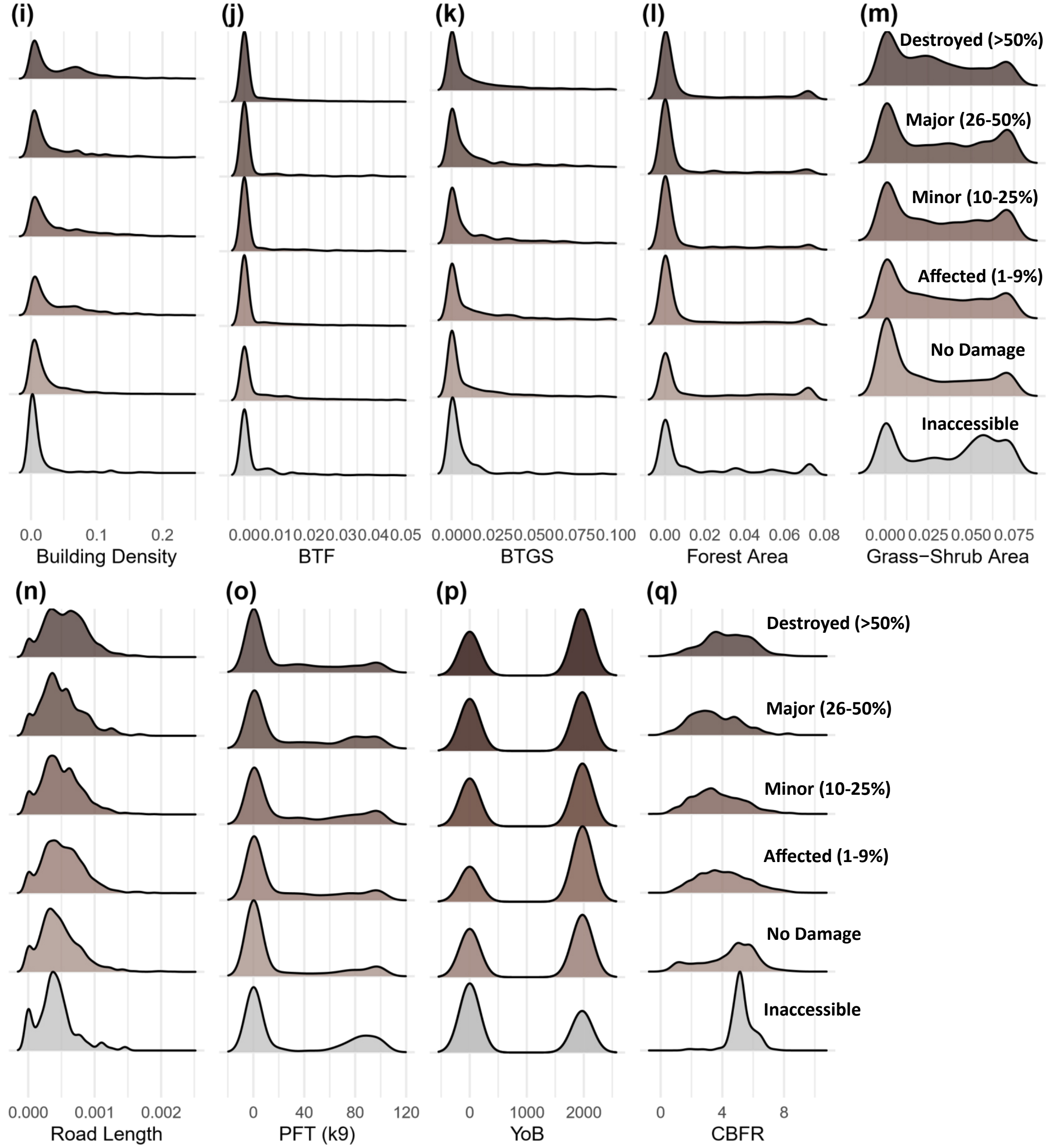

Figure S2: Probability density distribution of the different wildfire building damage across topography, ecosystems, human footprint, and building flammability in different damage classes from DINs observations.

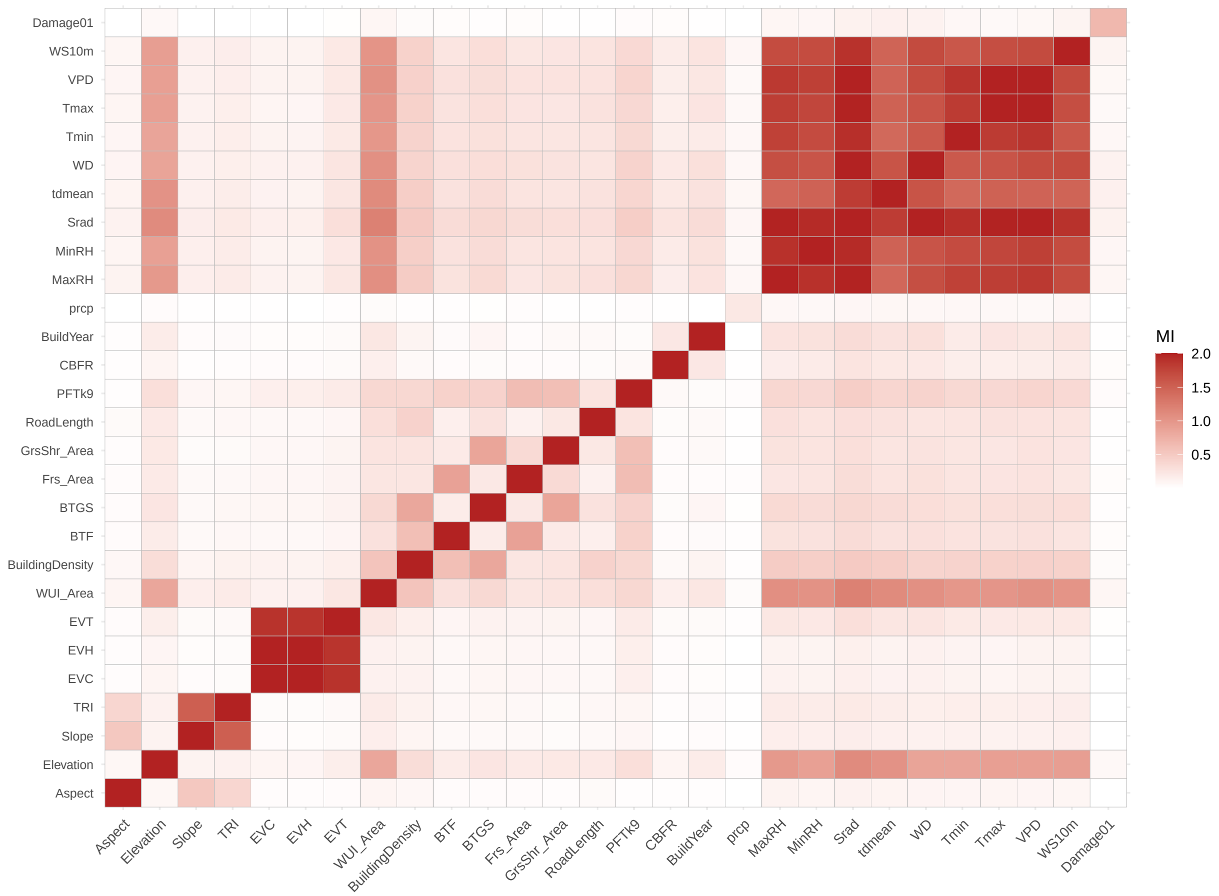

Figure S3: Mutual Information (MI) among the groups of the variables [Damage01: binary building damage classes, 0: No-damage, 1: Damaged].

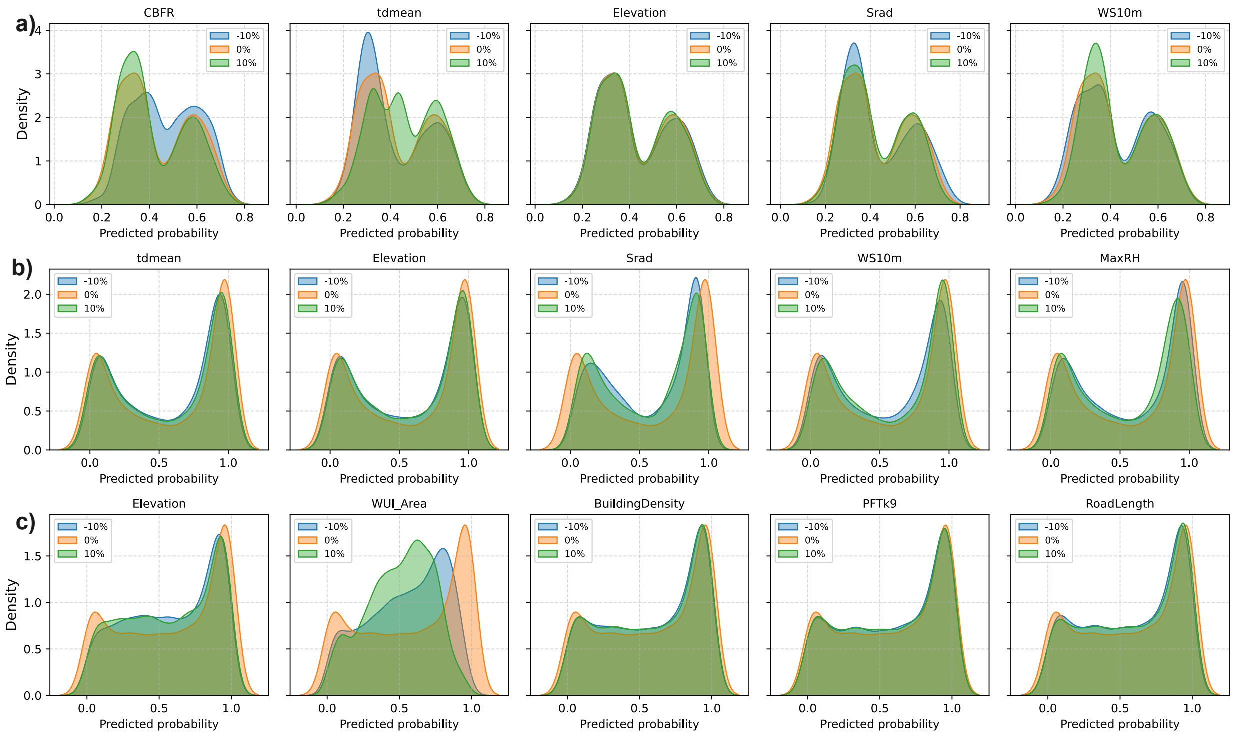

Figure S4: Sensitivity of Random Forest predictions to the 5 highest contributing features. The Rows correspond to three different models, Comprehensive (a), Enviro-Weather Hybrid (b), and Environmental Exposure (c), and columns show the top five features ranked by MDA. Predicted probabilities were computed after perturbing each feature −10%, 0%, and +10%. Kernel density illustrates the influence of feature perturbations on model predictions.

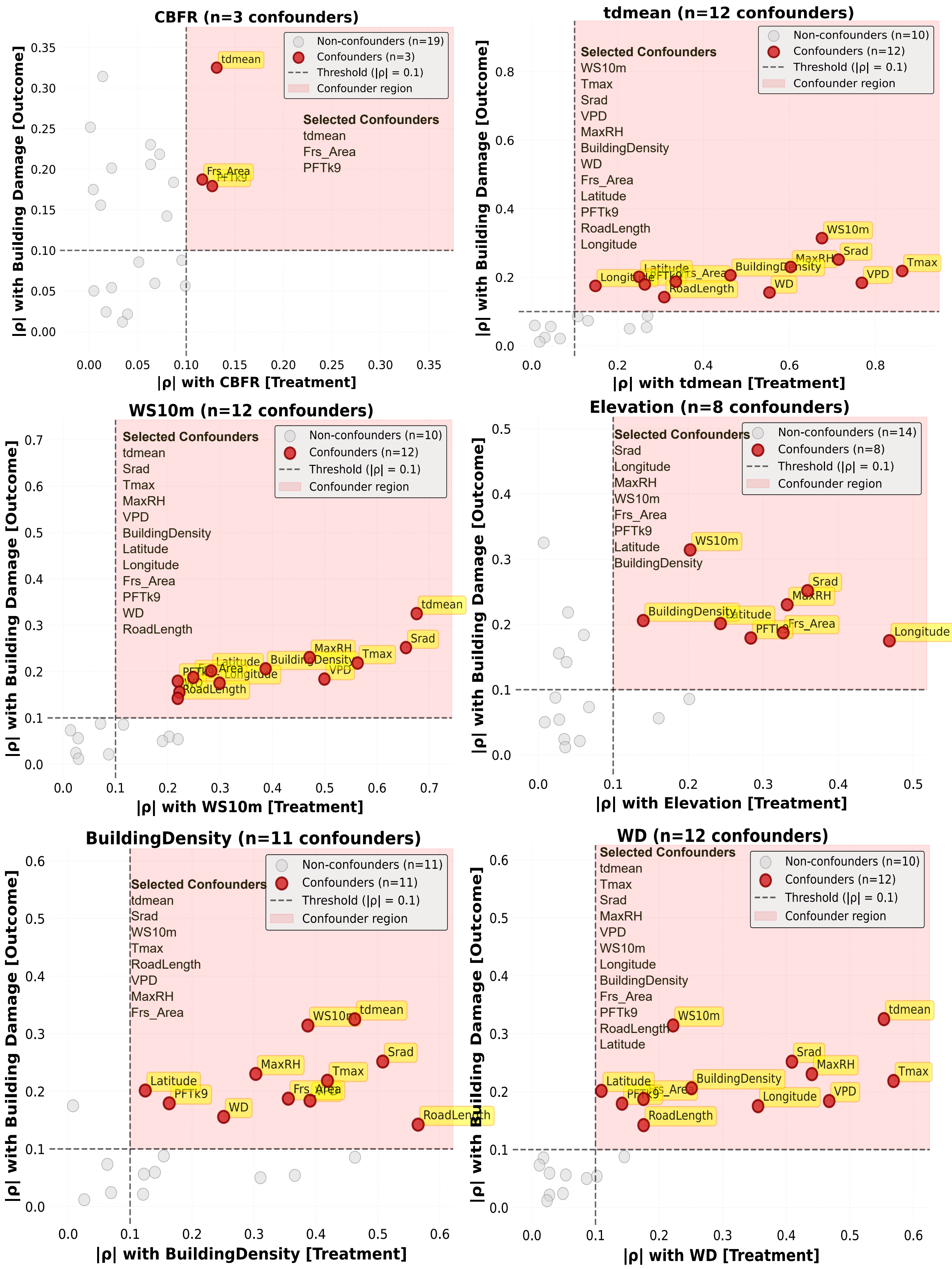
CBFR (n=3 confounders)
tdmean (n=12 confounders)
WS10m (n=12 confounders)
Elevation (n=8 confounders)
BuildingDensity (n=11 confounders)
WD (n=12 confounders)
|ρ| with Building Damage [Outcome]
|ρ| with CBFR [Treatment]
|ρ| with tdmean [Treatment]
|ρ| with WS10m [Treatment]
|ρ| with Elevation [Treatment]
|ρ| with BuildingDensity [Treatment]
|ρ| with WD [Treatment]
Non-confounders (n=19)
Confounders (n=3)
Threshold (|ρ| = 0.1)
Confounder region
Selected Confounders
tdmean
Frs_Area
PFTk9

Figure S5

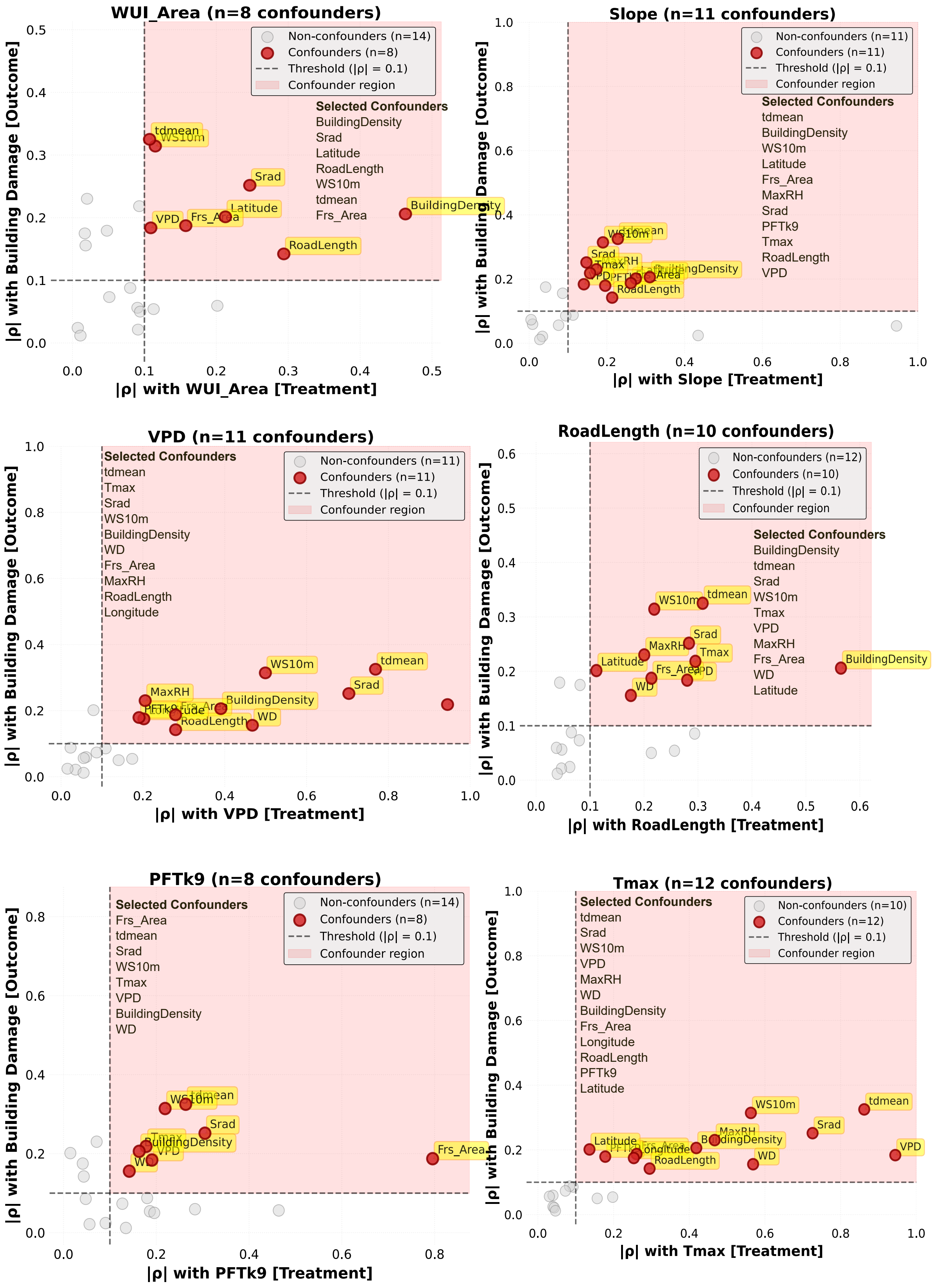
WUI_Area (n=8 confounders)
Non-confounders (n=14)
Confounders (n=8)
Threshold (|ρ| = 0.1)
Confounder region
Selected Confounders
BuildingDensity
Srad
Latitude
RoadLength
WS10m
tdmean
Frs_Area
|ρ| with Building Damage [Outcome]
|ρ| with WUI_Area [Treatment]
Slope (n=11 confounders)
Non-confounders (n=11)
Confounders (n=11)
Threshold (|ρ| = 0.1)
Confounder region
Selected Confounders
tdmean
BuildingDensity
WS10m
Latitude
Frs_Area
MaxRH
Srad
PFTk9
Tmax
RoadLength
VPD
|ρ| with Slope [Treatment]
VPD (n=11 confounders)
Selected Confounders
tdmean
Tmax
Srad
WS10m
BuildingDensity
WD
Frs_Area
MaxRH
RoadLength
Longitude
Non-confounders (n=11)
Confounders (n=11)
Threshold (|ρ| = 0.1)
Confounder region
|ρ| with VPD [Treatment]
RoadLength (n=10 confounders)
Non-confounders (n=12)
Confounders (n=10)
Threshold (|ρ| = 0.1)
Confounder region
Selected Confounders
BuildingDensity
tdmean
Srad
WS10m
Tmax
VPD
MaxRH
Frs_Area
WD
Latitude
|ρ| with RoadLength [Treatment]
PFTk9 (n=8 confounders)
Selected Confounders
Frs_Area
tdmean
Srad
WS10m
Tmax
VPD
BuildingDensity
WD
Non-confounders (n=14)
Confounders (n=8)
Threshold (|ρ| = 0.1)
Confounder region
|ρ| with PFTk9 [Treatment]
Tmax (n=12 confounders)
Selected Confounders
tdmean
Srad
WS10m
VPD
MaxRH
WD
BuildingDensity
Frs_Area
Longitude
RoadLength
PFTk9
Latitude
Non-confounders (n=10)
Confounders (n=12)
Threshold (|ρ| = 0.1)
Confounder region
|ρ| with Tmax [Treatment]

Figure S5

Figure S5: Systematic confounder selection for causal effect estimation using a dual-correlation criterion. For each treatment variable, potential confounders were assessed by computing Spearman rank correlations with both the treatment and the binary outcome (building damage). Variables satisfying $|\rho_{\text{treatment}}| \geq 0.1$ and $|\rho_{\text{outcome}}| \geq 0.1$ with p-values < 0.05 were classified as confounders (red), following VanderWeele (*76*). The x-axis shows $|\rho|$ with the treatment, and the y-axis shows $|\rho|$ with the outcome (Building Damage). The shaded quadrant indicates the confounder region where both criteria are met. Gray points represent variables excluded from the confounder set.

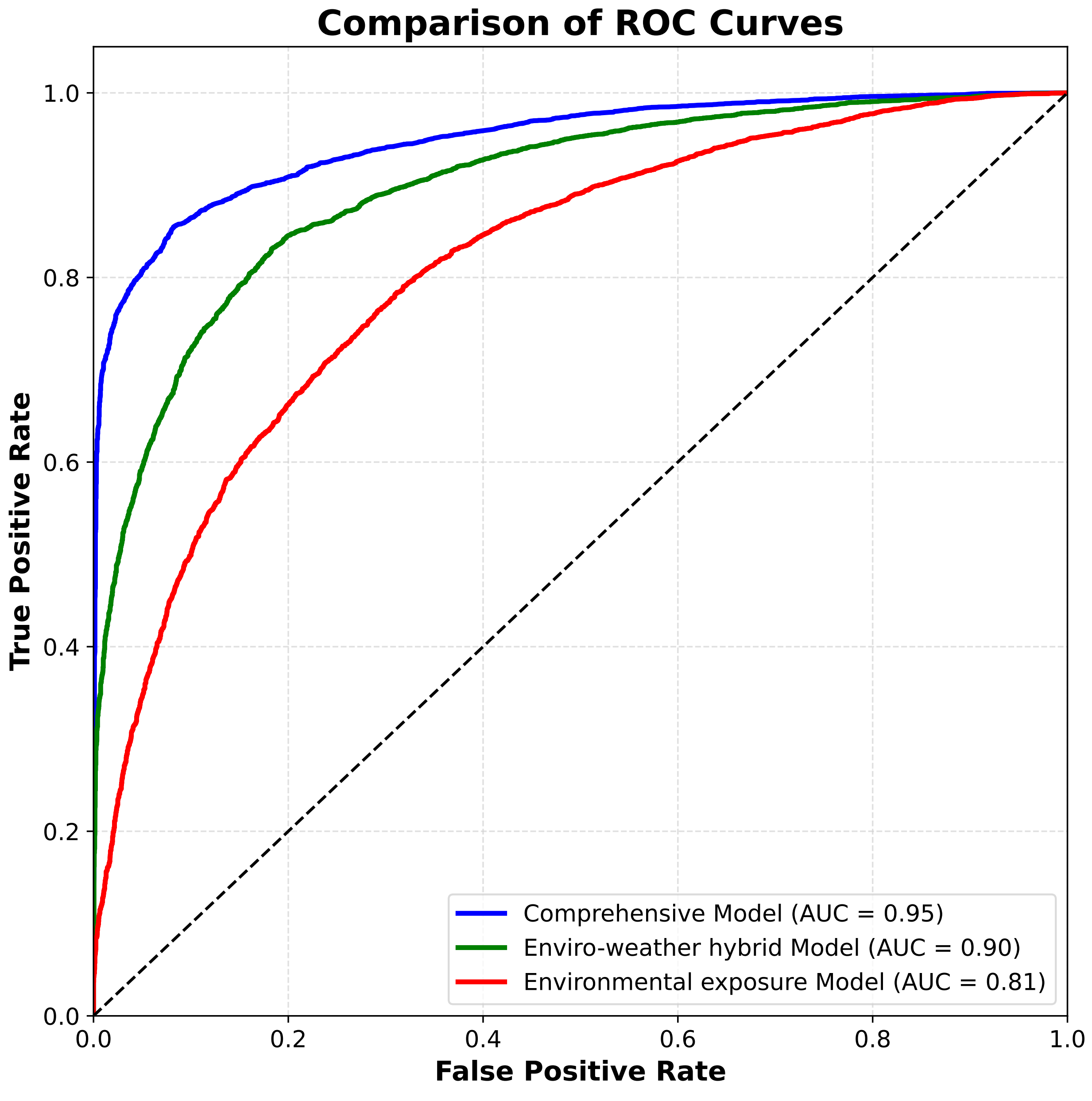

Figure S6: Receiver Operating Characteristic (ROC) curves and Area Under the Curve (AUC) values for three Random Forest models.

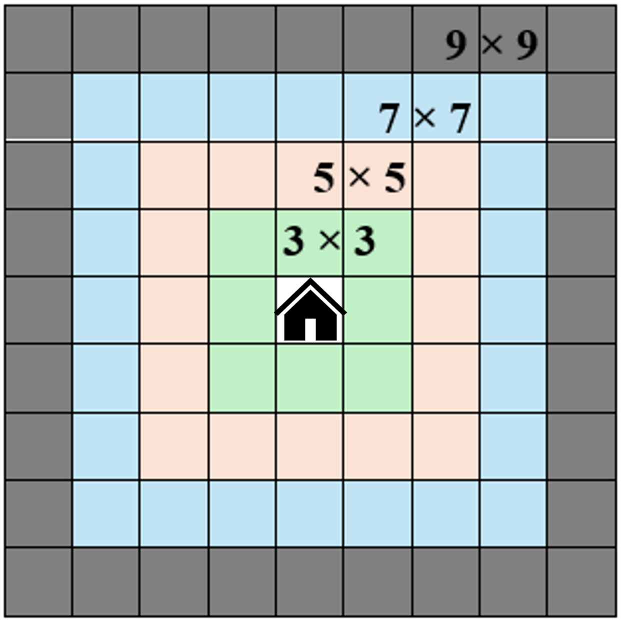

Figure S7: Different kernel sizes were used to estimate the proportion of fine and woody fuels surrounding each building, based on the 30 m National Land Cover Dataset, to explain building damage.

Table S1: Fire Names and their numbers of observations between 2013 and 2024, Cal-Fire (*11*).

Fire Name	Number of Observations
August Complex North	2
August Complex South	102
August Complex West	161
Bear	3205
Carmel	248
Castle	803
CZU Lightning Complex	4820
Doe	52
Elkhorn	40
Fire Name Not-Defined	79064
Hennessey	4243
Lassen	31
Mendocino Complex	319
Meyers	39
Napa	2673
Quail	59
River	341
Santa Rosa	633
SCU Lightning Complex	954
Sonoma	1352
St. Helena	109
Walbridge	839
Wind Complex	141

Table S2: Eigenvalues and explained variance of the 27 principal components.

PC	Eigenvalue	Variance (%)	Cumulative (%)
PC1	6.84	25.35	25.35
PC2	2.56	9.47	34.81
PC3	2.33	8.61	43.43
PC4	1.94	7.17	50.59
PC5	1.74	6.43	57.02
PC6	1.40	5.20	62.22
PC7	1.22	4.53	66.75
PC8	0.98	3.65	70.40
PC9	0.95	3.53	73.92
PC10	0.91	3.37	77.30
PC11	0.86	3.20	80.50
PC12	0.84	3.11	83.60
PC13	0.77	2.83	86.44
PC14	0.74	2.75	89.19
PC15	0.65	2.41	91.60
PC16	0.47	1.73	93.33
PC17	0.42	1.56	94.90
PC18	0.37	1.37	96.26
PC19	0.27	1.01	97.27
PC20	0.22	0.82	98.09
PC21	0.20	0.72	98.81
PC22	0.16	0.61	99.42
PC23	0.07	0.27	99.69
PC24	0.05	0.17	99.87
PC25	0.02	0.08	99.95
PC26	0.01	0.04	99.99
PC27	0.00	0.01	100.00

Table S3: Average feature contribution from SHAP for three models (Top 15 Features).

Comprehensive model	
**Feature**	**Contribution (%)**
CBFR	21.40
tdmean	16.20
WD	10.64
Srad	9.34
Elevation	8.10
WS10m	6.90
MaxRH	5.57
Tmax	5.41
VPD	4.05
TRI	3.37
WUI_Area	2.73
PFT_{k9}	2.57
BuildingDensity	2.00
RoadLength	1.10
Aspect	0.61

Table S3: Average feature contribution from SHAP (continued).

Enviro-weather hybrid model	
**Feature**	**Contribution (%)**
tdmean	20.25
WD	12.00
Elevation	11.31
Srad	10.02
Tmax	8.32
WS10m	6.97
VPD	5.91
MaxRH	5.53
TRI	4.23
PFT_{k9}	4.20
WUI_Area	3.33
BuildingDensity	3.13
RoadLength	2.02
Slope	1.64
Aspect	1.15

Table S3: Average feature contribution from SHAP (continued).

Environmental exposure model	
**Feature**	**Contribution (%)**
Elevation	22.98
WUI_Area	15.32
PFT_{k9}	10.02
BuildYear	9.32
TRI	8.20
Frs_Area	7.67
EVH	5.69
RoadLength	4.33
BuildingDensity	4.18
EVT	4.09
BTF	3.64
Slope	2.39
Aspect	2.17

Table S4: Confusion Matrices of building damage and no-damage binary classification.

**Model**	**Actual \ Predicted**	**No Damage (0)**	**Damage (1)**
Comprehensive	No Damage (0)	7,694	514
	Damage (1)	889	10,949
Enviro-Weather Hybrid	No Damage (0)	7,215	993
	Damage (1)	1,105	10,733
Environmental Exposure	No Damage (0)	6,728	1,480
	Damage (1)	1,228	10,610

Table S5: Fire-wise performance of models.

Fire Name	Comprehensive	Enviro-Weather Hybrid	Environmental Exposure
**Overall Accuracy**			
CZU Lightning Complex	0.77	0.65	0.53
Hennessey	0.82	0.67	0.56
Bear	0.76	0.76	0.46
**Weighted Precision**			
CZU Lightning Complex	0.82	0.70	0.57
Hennessey	0.83	0.60	0.58
Bear	0.72	0.66	0.65
**Weighted F1-score**			
CZU Lightning Complex	0.74	0.52	0.54
Hennessey	0.81	0.56	0.57
Bear	0.72	0.68	0.50

Table S6: Referenced studies that examined building fire risk as a function of material composition and wildfire exposure conditions.

**Building Structure/Material**	**Finding on Flammability**	**Reference**
Wood siding and decks	Easily ignited by embers; major cause of structural ignition during wildfires	(*41, 79*)
Single-pane windows	Break quickly under radiant heat, allowing ember entry	(*80*)
Double-pane tempered glass windows	Provide better thermal resistance to heat and embers	(*79*)
Open eaves and soffits	Act as entry points for embers, increasing ignition risk	(*28, 81*)
Enclosed eaves	Reduce vulnerability to ember intrusion	(*79*)
Class A roof (e.g., asphalt composition, metal)	Resistant to fire spread and ember ignition	(*82*)
Wood shake roofs (untreated)	Extremely flammable and often banned in high-risk areas	(*41, 83*)
Ember-resistant vents	Reduce likelihood of embers entering attics and crawlspaces	(*79, 84*)
Combustible fencing attached to homes	Can act as a wick, carrying fire to structures	(*79, 85*)
Decks with open undersides	Highly vulnerable to radiant heat and ember accumulation	(*85*)

Table S6: Referenced studies that examined building fire risk (continued).

**Building Structure/Material**	**Finding on Flammability**	**Reference**
Ignition-resistant deck materials	Reduce structural vulnerability, especially with closed substructures	(*84*)
Mobile homes (single/double wide)	More flammable due to combustible materials and poor defensible space	(*85*)
Metal screens	Effective at blocking embers from entering vents or other openings	(*86*)
Vegetation near structures	Increases radiant heat exposure and ember accumulation	(*12, 41*)
Radiant barriers	Help reduce internal heating, but do not prevent direct flame contact damage	(*87*)
Garage doors without weather stripping	Allow embers and heat entry into interior spaces	(*85*)
Small mesh screens on attic vents	Significantly reduce ember penetration while maintaining ventilation	(*79*)
Flammable mulch near walls	Can ignite from embers and spread fire to siding	(*41, 88*)
Fire-resistant landscaping	Helps create defensible space and reduce fire spread toward structures	(*89*)

Table S7: Comparison of Model Performance Across 10-fold Cross-validation (OA and Macro F1)

Fold	Comprehensive		Enviro-weather hybrid		Environmental exposure	
	**OA**	**F1**	**OA**	**F1**	**OA**	**F1**
1	0.879	0.892	0.832	0.852	0.741	0.780
2	0.879	0.893	0.824	0.846	0.737	0.778
3	0.875	0.886	0.819	0.833	0.740	0.777
4	0.888	0.903	0.837	0.858	0.747	0.790
5	0.877	0.890	0.829	0.847	0.751	0.791
6	0.875	0.891	0.830	0.852	0.739	0.781
7	0.880	0.893	0.823	0.841	0.748	0.786
8	0.878	0.893	0.830	0.850	0.752	0.793
9	0.886	0.900	0.837	0.856	0.747	0.786
10	0.879	0.893	0.827	0.849	0.746	0.786
Mean	0.880	0.893	0.829	0.848	0.745	0.785
SD	0.004	0.005	0.006	0.007	0.005	0.006

REFERENCES

1. H. Buechi, P. Weber, S. Heard, D. Cameron, A. J. Plantinga, Long-term trends in wildfire damages in California. *Int. J. Wildland Fire* **30**, 757–762 (2021).

2. S. Li, T. Banerjee, Spatial and temporal pattern of wildfires in California from 2000 to 2019. *Sci. Rep.* **11**, 8779 (2021).

3. V. C. Radeloff, D. P. Helmers, H. A. Kramer, M. H. Mockrin, P. M. Alexandre, A. Bar-Massada, V. Butsic, T. J. Hawbaker, S. Martinuzzi, A. D. Syphard, S. I. Stewart, Rapid growth of the US wildland-urban interface raises wildfire risk. *Proc. Natl. Acad. Sci. U.S.A.* **115**, 3314–3319 (2018).

4. T. Schoennagel, J. K. Balch, H. Brenkert-Smith, P. E. Dennison, B. J. Harvey, M. A. Krawchuk, N. Mietkiewicz, P. Morgan, M. A. Moritz, R. Rasker, M. G. Turner, C. Whitlock, Adapt to more wildfire in western North American forests as climate changes. *Proc. Natl. Acad. Sci. U.S.A.* **114**, 4582–4590 (2017).

5. M. Chalabi, More than 80% of new California properties are in high fire-risk areas, www.theguardian.com/news/datablog/2025/feb/15/california-wildfire-building-risk (2025) [accessed 6 May 2025].

6. J. Boomhower, Adapting to growing wildfire property risk. *Science* **382**, 638–641 (2023).

7. M. Papathoma-Köhle, M. Schlögl, C. Garlichs, M. Diakakis, S. Mavroulis, S. Fuchs, A wildfire vulnerability index for buildings. *Sci. Rep.* **12**, 6378 (2022).

8. S. Dossi, B. Messerschmidt, L. M. Ribeiro, M. Almeida, G. Rein, Relationships between building features and wildfire damage in California, USA and Pedrógão Grande, Portugal. *Int. J. Wildland Fire* **32**, 296–312 (2023).

9. H. A. Kramer, M. H. Mockrin, P. M. Alexandre, V. C. Radeloff, High wildfire damage in interface communities in California. *Int. J. Wildland Fire* **28**, 641–650 (2019).

10. V. C. Radeloff, M. H. Mockrin, D. Helmers, A. Carlson, T. J. Hawbaker, S. Martinuzzi, F. Schug, P. M. Alexandre, H. A. Kramer, A. M. Pidgeon, Rising wildfire risk to houses in the United States, especially in grasslands and shrublands. *Science* **382**, 702–707 (2023).

11. CAL FIRE, CAL FIRE Damage Inspection (DINS) Data, https://hub-calfire-forestry.hub.arcgis.com/datasets/cal-fire-damage-inspection-dins-data/about (2025) [accessed 6 May 2025].

12. A. D. Syphard, J. E. Keeley, A. B. Massada, T. J. Brennan, V. C. Radeloff, Housing arrangement and location determine the likelihood of housing loss due to wildfire. *PLOS ONE* **7**, e33954 (2012).

13. J. M. Kodero, B. S. Felzer, Y. Shi, Future transition from forests to shrublands and grasslands in the western United States is expected to reduce carbon storage. *Commun. Earth Environ.* **5**, 78 (2024).

14. S. Fares, S. Bajocco, L. Salvati, N. Camarretta, J.-L. Dupuy, G. Xanthopoulos, M. Guijarro, J. Madrigal, C. Hernando, P. Corona, Characterizing potential wildland fire fuel in live vegetation in the Mediterranean region. *Ann. For. Sci.* **74**, 1 (2017).

15. Y. Michael, G. Kozokaro, S. Brenner, I. M. Lensky, Improving WRF-fire wildfire simulation accuracy using SAR and time series of satellite-based vegetation indices. *Remote Sens.* **14**, 2941 (2022).

16. A. Papalou, D. K. Baros, Assessing structural damage after a severe wildfire: A case study. *Buildings* **9**, 171 (2019).

17. A. Edalati-nejad, M. Ghodrat, J. J. Sharples, On the interaction of wind, fire intensity and downslope terrain with implications for building standards in wildfire-prone areas. *Int. J. Wildland Fire* **32**, 1619–1632 (2023).

18. M. Kumar, S. Li, P. Nguyen, T. Banerjee, Examining the existing definitions of wildland-urban interface for California. *Ecosphere* **13**, e4306 (2022).

19. B. J. Meacham, D. Charters, P. Johnson, M. Salisbury, “Building fire risk analysis” in *SFPE Handbook of Fire Protection Engineering* (Springer, New York, NY, 2016), pp. 2941–2991.

20. S. L. Manzello, S. Suzuki, Y. Hayashi, Enabling the study of structure vulnerabilities to ignition from wind driven firebrand showers: A summary of experimental results. *Fire Saf. J.* **54**, 181–196 (2012).

21. Y. Jin, J. T. Randerson, N. Faivre, S. Capps, A. Hall, M. L. Goulden, Contrasting controls on wildland fires in Southern California during periods with and without Santa Ana winds. *J. Geophys. Res. Biogeo.* **119**, 432–450 (2014).

22. California Department of Forestry and Fire Protection, 2025 Fire Season Incident Archive, www.fire.ca.gov/incidents/2025 (2025) [accessed 29 April 2025].

23. M. Qiu, D. Chen, M. Kelp, J. Li, G. Huang, M. D. Yazdi, The rising threats of wildland-urban interface fires in the era of climate change: The Los Angeles 2025 fires. *Int. J. Hydrogen Energ.* **6**, 100835 (2025).

24. S. T. Seydi, Assessment of the January 2025 Los Angeles County wildfires: A multi-modal analysis of impact, response, and population exposure. arXiv:2501.17880 [eess.SP] (2025).

25. R. S. Hakes, S. E. Caton, D. J. Gorham, M. J. Gollner, A review of pathways for building fire spread in the wildland urban interface part II: Response of components and systems and mitigation strategies in the United States. *Fire Technol.* **53**, 475–515 (2017).

26. L. M. Ribeiro, A. Rodrigues, D. Lucas, D. X. Viegas, The impact on structures of the Pedrógão Grande fire complex in June 2017 (Portugal). *Fire* **3**, 57 (2020).

27. A. D. Syphard, J. E. Keeley, Factors associated with structure loss in the 2013–2018 California wildfires. *Fire* **2**, 49 (2019).

28. A. D. Syphard, T. J. Brennan, J. E. Keeley, The importance of building construction materials relative to other factors affecting structure survival during wildfire. *Int. J. Disaster Risk Reduct.* **21**, 140–147 (2017).

29. M. Reichstein, G. Camps-Valls, B. Stevens, M. Jung, J. Denzler, N. Carvalhais, F. Prabhat, Deep learning and process understanding for data-driven Earth system science. *Nature* **566**, 195–204 (2019).

30. F. Abid, A survey of machine learning algorithms based forest fires prediction and detection systems. *Fire Technol.* **57**, 559–590 (2021).

31. Y. Ban, P. Zhang, A. Nascetti, A. R. Bevington, M. A. Wulder, Near real-time wildfire progression monitoring with Sentinel-1 SAR time series and deep learning. *Sci. Rep.* **10**, 1322 (2020).

32. M. Galanis, K. Rao, X. Yao, Y.-L. Tsai, J. Ventura, G. A. Fricker, DamageMap: A post-wildfire damaged buildings classifier. *Int. J. Disaster Risk Reduct.* **65**, 102540 (2021).

33. A. M. Braik, M. Koliou, Automated building damage assessment and large-scale mapping by integrating satellite imagery, GIS, and deep learning. *Comput. Aided Civ. Inf. Eng.* **39**, 2389–2404 (2024).

34. S. Feuerriegel, D. Frauen, V. Melnychuk, J. Schweisthal, K. Hess, A. Curth, S. Bauer, N. Kilbertus, I. S. Kohane, M. van der Schaar, Causal machine learning for predicting treatment outcomes. *Nat. Med.* **30**, 958–968 (2024).

35. P. M. Alexandre, S. I. Stewart, M. H. Mockrin, N. S. Keuler, A. D. Syphard, A. Bar-Massada, M. K. Clayton, V. C. Radeloff, The relative impacts of vegetation, topography and spatial arrangement on building loss to wildfires in case studies of California and Colorado. *Landsc. Ecol.* **31**, 415–430 (2016).

36. A. Chulahwat, H. Mahmoud, S. Monedero, F. J. Diez Vizcaíno, J. Ramirez, D. Buckley, A. C. Forradellas, Integrated graph measures reveal survival likelihood for buildings in wildfire events. *Sci. Rep.* **12**, 15954 (2022).

37. M. Zamanialaei, D. San Martin, M. Theodori, D. M. J. Purnomo, A. Tohidi, C. Lautenberger, Y. Qin, A. Trouvé, M. Gollner, Fire risk to structures in California's Wildland-Urban Interface. *Nat. Commun.* **16**, 8041 (2025).

38. S. Li, V. Dao, M. Kumar, P. Nguyen, T. Banerjee, Mapping the wildland-urban interface in California using remote sensing data. *Sci. Rep.* **12**, 5789 (2022).

39. K. A. McKinnon, A. Poppick, I. R. Simpson, Hot extremes have become drier in the United States Southwest. *Nat. Clim. Chang.* **11**, 598–604 (2021).

40. J. D. Cohen, Preventing disaster: Home ignitability in the wildland-urban interface. *J. For.* **98**, 15–21 (2000).

41. L. Dong, L. R. Leung, Y. Qian, Y. Zou, F. Song, X. Chen, Meteorological environments associated with California wildfires and their potential roles in wildfire changes during 1984–2017. *J. Geophys. Res. Atmos.* **126**, e2020JD033180 (2021).

42. W. Zhang, S. S. Wang, Y. Chikamoto, R. Gillies, M. LaPlante, V. Hari, A weather pattern responsible for increasing wildfires in the western United States. *Environ. Res. Lett.* **20**, 014007 (2025).

43. K. Guirguis, A. Gershunov, B. Hatchett, T. Shulgina, M. J. DeFlorio, A. C. Subramanian, J. Guzman-Morales, R. Aguilera, R. Clemesha, T. W. Corringham, L. Delle Monache, D. Reynolds, A. Tardy, I. Small, F. M. Ralph, Winter wet–dry weather patterns driving atmospheric rivers and Santa Ana winds provide evidence for increasing wildfire hazard in California. *Climate Dynam.* **60**, 1729–1749 (2023).

44. E. E. Knapp, A. A. Bernal, J. M. Kane, C. J. Fettig, M. P. North, Variable thinning and prescribed fire influence tree mortality and growth during and after a severe drought. *For. Ecol. Manage.* **479**, 118595 (2021).

45. E. E. Knapp, R. L. Carlson, M. P. North, J. L. Lydersen, B. M. Collins, "Restoring forest heterogeneity with thinning and prescribed fire: Initial results from the central Sierra Nevada, California" in *The 2019 National Silviculture Workshop: A Focus on Forest Management-Research Partnerships*, L. S. Pile, R. L. Deal, D. C. Dey, D. Gwaze, J. M. Kabrick; B. J. Palik; T. M. Schuler, comps. (Gen. Tech. Rep. NRS-P-193, US Department of Agriculture, Forest Service, Northern Research Station, 2020), pp. 216–226.

46. California Board of Forestry and Fire Protection, Defensible Space Zones 0, 1, and 2 (2025), https://bof.fire.ca.gov/projects-and-programs/defensible-space-zones-0-1-and-2 [accessed 31 Jan 2025].

47. California Legislature, Senate Bill No. 504 (2023–2024) (2024), https://leginfo.legislature.ca.gov/faces/billNavClient.xhtml?bill_id=202320240SB504 [accessed 31 Jan. 2025].

48. K. M. Yedinak, E. K. Strand, J. K. Hiers, J. M. Varner, Embracing complexity to advance the science of wildland fire behavior. *Fire* **1**, 20 (2018).

49. M. Goss, D. L. Swain, J. T. Abatzoglou, A. Sarhadi, C. A. Kolden, A. P. Williams, N. S. Diffenbaugh, Climate change is increasing the likelihood of extreme autumn wildfire conditions across California. *Environ. Res. Lett.* **15**, 094016 (2020).

50. I. W. Park, M. L. Mann, L. E. Flint, A. L. Flint, M. Moritz, Relationships of climate, human activity, and fire history to spatiotemporal variation in annual fire probability across California. *PLOS ONE* **16**, e0254723 (2021).

51. A. D. Syphard, H. Rustigian-Romsos, J. E. Keeley, Multiple-scale relationships between vegetation, the wildland–urban interface, and structure loss to wildfire in California. *Fire* **4**, 12 (2021).

52. S. Li, V. Dao, M. Kumar, N. Phu, T. Banerjee, Wildland-urban interface in California using remote sensing data, Dryad (2022); https://doi.org/10.7280/D1B98J.

53. Microsoft, GlobalMLBuildingFootprints, https://github.com/microsoft/GlobalMLBuildingFootprints (2025), original work published 2022 [accessed 6 May 2025].

54. U.S. Census Bureau, 2024 TIGER/Line Shapefiles, www.census.gov/geographies/mapping-files/time-series/geo/tiger-line-file.html (2024) [accessed 6 May 2025].

55. LANDFIRE Program, LANDFIRE: Landscape Fire and Resource Management Planning Tools, www.landfire.gov/ (2025) [accessed 6 May 2025].

56. U.S. Geological Survey, National Land Cover Database, https://www.usgs.gov/centers/eros/science/national-land-cover-database (2020) [accessed 6 May 2025.

57. L. Holsinger, S. A. Parks, C. Miller, Weather, fuels, and topography impede wildland fire spread in western US landscapes. *For. Ecol. Manage.* **380**, 59–69 (2016).

58. California Department of Forestry and Fire Protection, CAL FIRE Incidents, www.fire.ca.gov/incidents/ (2025) [accessed 6 May 2025].

59. C. Daly, Descriptions of PRISM spatial climate datasets for the conterminous United States (PRISM Climate Group, Oregon State University, 2013), pp. 1–14.

60. J. T. Abatzoglou, Development of gridded surface meteorological data for ecological applications and modelling. *Int. J. Climatol.* **33**, 121–131 (2013).

61. E. ASTM, Standard Test Method for Surface Burning Characteristics of Building Materials (84) (ASTM International, 2014).

62. M. B. Kursa, A. Jankowski, W. R. Rudnicki, Boruta–a system for feature selection. *Fundam. Inform.* **101**, 271–285 (2010).

63. D. McKenzie, C. Miller, D. A. Falk, *The Landscape Ecology of Fire*, vol. 213 (Springer Science & Business Media, 2011).

64. P. E. McKight, J. Najab, Kruskal-Wallis test. *The Corsini Encyclopedia of Psychology* (John Wiley & Sons, Inc., 2010), pp. 1–1.

65. H. Abdi, L. J. Williams, Principal component analysis. *Wiley Interdiscip. Rev. Comput. Stat.* **2**, 433–459 (2010).

66. A. Kraskov, H. Stögbauer, P. Grassberger, Estimating mutual information. *Phys. Rev. E* **69**, 066138 (2004).

67. I. Cohen, Y. Huang, J. Chen, J. Benesty, J. Benesty, J. Chen, Y. Huang, I. Cohen, "Pearson correlation coefficient" in *Noise Reduction in Speech Processing* (Springer Berlin, Heidelberg, 2009), pp. 1–4.

68. N. V. Chawla, K. W. Bowyer, L. O. Hall, W. P. Kegelmeyer, SMOTE: Synthetic minority over-sampling technique. *J Artif Intell Res* **16**, 321–357 (2002).

69. L. Breiman, Random forests. *Mach. Learn.* **45**, 5–32 (2001).

70. S. Bar, B. R. Parida, A. C. Pandey, B. U. Shankar, P. Kumar, S. K. Panda, M. D. Behera, Modeling and prediction of fire occurrences along an elevational gradient in Western Himalayas. *Appl. Geogr.* **151**, 102867 (2023).

71. S. M. Lundberg, G. Erion, H. Chen, A. DeGrave, J. M. Prutkin, B. Nair, R. Katz, J. Himmelfarb, N. Bansal, S.-I. Lee, From local explanations to global understanding with explainable AI for trees. *Nature machine intelligence* **2**, 56–67 (2020).

72. Y. Nohara, K. Matsumoto, H. Soejima, N. Nakashima, Explanation of machine learning models using shapley additive explanation and application for real data in hospital. *Comput. Methods Programs Biomed.* **214**, 106584 (2022).

73. L. Wang, B. Chen, J. Ouyang, Y. Mu, L. Zhen, L. Yang, W. Xu, L. Tang, Causal-inference machine learning reveals the drivers of China's 2022 ozone rebound. *Environ. Sci. Ecotechnol.* **24**, 100524 (2025).

74. V. Sitokonstantinou, E. Diaz, J. Cerda-Bautista, M. Piles, I. N. Athanasiadis, I. Tsoumas, G. Camps-Valls, Nature mediLearning for sustainable agriculture, in *NeurIPS 2025 Workshop on CauScien: Uncovering Causality in Science* (2025).

75. V. Sitokonstantinou, E. D. S. Porras, J. C. Bautista, M. Piles, I. Athanasiadis, H. Kerner, G. Martini, L. belle Sweet, I. Tsoumas, J. Zscheischler, G. Camps-Valls, Causal machine learning for sustainable agroecosystems. arXiv:2408.13155 [cs.LG] (2024).

76. T. J. VanderWeele, Principles of confounder selection. *Eur. J. Epidemiol.* **34**, 211–219 (2019).

77. K. Battocchi, E. Dillon, M. Hei, G. Lewis, P. Oka, M. Oprescu, V. Syrgkaniss, EconML: A Python package for ML-based heterogeneous treatment effects estimation, version 0.x (2019); https://github.com/py-why/EconML.

78. Q. Kang, X. Song, X. Xin, B. Chen, Y. Chen, X. Ye, B. Zhang, Machine learning-aided causal inference framework for environmental data analysis: A COVID-19 case study. *Environ. Sci. Technol.* **55**, 13400–13410 (2021).

79. S. L. Quarles, Y. Valachovic, G. M. Nakamura, G. A. Nader, M. J. De Lasaux, Home survival in wildfire-prone areas: Building materials and design considerations (University of California Agriculture and Natural Resources (UC ANR), 2010).

80. S. L. Manzello, S. Suzuki, D. Nii, Full-scale experimental investigation to quantify building component ignition vulnerability from mulch beds attacked by firebrand showers. *Fire Technol.* **53**, 535–551 (2017).

81. A. Maranghides, W. Mell, A case study of a community affected by the Witch and Guejito wildland fires. *Fire Technol.* **47**, 379–420 (2011).

82. R. Willey, National fire protection association codes and standards. *Chem. Eng. Prog.* **108**, 4–4 (2012).

83. A. D. Syphard, T. J. Brennan, J. E. Keeley, The role of defensible space for residential structure protection during wildfires. *Int. J. Wildland Fire* **23**, 1165–1175 (2014).

84. K. Barrett, S. L. Quarles, Retrofitting homes: Cost of mitigation strategies for improved wildfire resistance. *Fire Mater.* , (2024).

85. C. Hand, *Engineering Solutions for Wildfires* (The Rosen Publishing Group Inc., 2019).

86. J. Hashempour, "Investigating potential of metal mesh to contain wildfires," thesis, University of Southern Queensland (2016).

87. F. Takahashi, Whole-house fire blanket protection from wildland-urban interface fires. *Front. Mech. Eng.* **5**, 60 (2019).

88. NFPA, *NFPA-1144: Standard for Reducing Structure Ignition Hazards from Wildland Fire* (2008 Edition) (National Fire Protection Association, (2008).

89. A. C. Fernandez-Pello, Wildland fire spot ignition by sparks and firebrands. *Fire Saf. J.* **91**, 2–10 (2017).
